# A DNA‐Modularized STING Agonist with Macrophage‐Selectivity and Programmability for Enhanced Anti‐Tumor Immunotherapy

**DOI:** 10.1002/advs.202400149

**Published:** 2024-06-19

**Authors:** Yingzhi Chen, Ruike Li, Qiao Duan, Lingling Wu, Xinyi Li, Aoxiang Luo, Yongming Zhang, Na Zhao, Kai Cui, Wenwei Wu, Tize Liu, Jian‐Bo Wan, Liufu Deng, Guiying Li, Lijun Hou, Weihong Tan, Zeyu Xiao

**Affiliations:** ^1^ Department of Pharmacology and Chemical Biology Key Laboratory of Cell Differentiation and Apoptosis of Chinese Ministry of Education Shanghai Jiao Tong University School of Medicine Shanghai 200025 China; ^2^ Institute of Molecular Medicine Shanghai Key Laboratory of Nucleic Acid Chemistry and Nanomedicine Renji Hospital Shanghai Jiao Tong University School of Medicine Shanghai 200127 China; ^3^ Shanghai Institute of Immunology Department of Immunology and Microbiology Shanghai Jiao Tong University School of Medicine Shanghai 200025 China; ^4^ State Key Laboratory of Quality Research in Chinese Medicine Institute of Chinese Medical Sciences University of Macau Taipa Macau 999078 China; ^5^ Department of Nephrology the Affiliated Hospital of Hebei Engineering University Hebei 056038 China; ^6^ Department of Neurosurgery Changzheng Hospital Naval Medical University Shanghai 200003 China

**Keywords:** aptamer, cancer immunotherapy, cGAS‐STING, DNA nanotechnology, drug delivery, tumor‐associated macrophages

## Abstract

The activation of cyclic GMP‐AMP (cGAMP) synthase (cGAS) and its adaptor, stimulator of interferon genes (STING), is known to reprogram the immunosuppressive tumor microenvironment for promoting antitumor immunity. To enhance the efficiency of cGAS‐STING pathway activation, macrophage‐selective uptake, and programmable cytosolic release are crucial for the delivery of STING agonists. However, existing polymer‐ or lipid‐based delivery systems encounter difficulty in integrating multiple functions meanwhile maintaining precise control and simple procedures. Herein, inspired by cGAS being a natural DNA sensor, a modularized DNA nanodevice agonist (DNDA) is designed that enable macrophage‐selective uptake and programmable activation of the cGAS‐STING pathway through precise self‐assembly. The resulting DNA nanodevice acts as both a nanocarrier and agonist. Upon local administration, it demonstrates the ability of macrophage‐selective uptake, endosomal escape, and cytosolic release of the cGAS‐recognizing DNA segment, leading to robust activation of the cGAS‐STING pathway and enhanced antitumor efficacy. Moreover, DNDA elicits a synergistic therapeutic effect when combined with immune checkpoint blockade. The study broadens the application of DNA nanotechnology as an immune stimulator for cGAS‐STING activation.

## Graphical abstract

A modularized DNA nanodevice agonist (DNDA) has been rationally designed and constructed as both a nanocarrier and agonist. DNA‐based modules with desired functions enable DNDA to accomplish macrophage‐selective uptake and programmable cGAS‐STING activation. By reversing the immunosuppressive tumor microenvironment, DNDA exhibits a synergistic therapeutic effect when combined with immune checkpoint inhibitors. The cell‐selective STING activation with biocompatibility makes it potential for clinical translation.

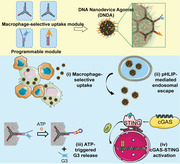

## 1 Introduction

The cGAS‐STING pathway plays a significant role in enhancing the immune response by activating innate immunity, and therefore has been recognized as the next‐generation cancer immunotherapy.^[^ ^1^ ^]^ In this pathway, cGAS binds to double‐stranded DNA (dsDNA), triggering a catalytic activity that results in the production of cGAMP, which then stimulates type I interferon (IFN‐I) responses.^[^ ^2^ ^]^ In addition, the binding of STING and cyclic dinucleotides (CDNs),^[^ ^3^ ^]^ as well as the endoplasmic reticulum  stress‐mediated STING shift^[^ ^4^ ^]^ are also ways to activate the cGAS‐STING pathway. To enhance the activation of the cGAS‐STING pathway, macrophage‐selective uptake, and programmable cytosolic release are crucial for the delivery of STING agonists. As a major proportion of innate immune cells in the tumor microenvironment, macrophages are considered as the ideal target for STING activation, by promoting antigen presentation, secreting proinflammatory cytokines, and increasing cytotoxic T lymphocyte infiltration.^[^ ^5^ ^]^ Non‐specific STING activation in normal tissues may cause unwanted inflammatory reactions,^[^ ^2^, ^6^ ^]^ whereas the cGAS‐STING pathway in tumor cells can be far less activated compared to that in macrophages.^[^ ^7^ ^]^ After being phagocyted by macrophages, STING agonists still need a programmable process for activation, including escape from endosomes, the release of agonists in the cytoplasm, and finally recognition by cGAS or STING for activation.^[^ ^8^ ^]^

In pursuit of achieving macrophage‐selectivity and programmability, current strategies mainly rely on polymer‐ or lipid‐based systems for STING agonist delivery.^[^ ^9^ ^]^ Besides, metal‐based delivery systems have also been utilized for cGAS‐STING activation,^[^ ^10^ ^]^ and small‐molecular STING agonists can be administered systemically or orally to enhance anti‐tumor immunity.^[^ ^11^ ^]^ The development of small‐molecular agonists involves the screening of compound libraries and chemical modification.^[^ ^11a^ ^]^ The construct of these delivery systems involves the screening of endosome‐disrupting materials, complex synthesis reactions, multistep formulation processes, and the modification of targeting ligands. Despite their effectiveness, these strategies face two major challenges. First, it is difficult to precisely regulate the formulation parameters, such as the polymerization degree, reaction efficiency, functional group modification, and encapsulation efficiency of the STING agonist. The lack of precise control can lead to poor batch‐to‐batch reproducibility, ultimately affecting therapeutic efficacy. Second, the synthesis and modification of these delivery systems are complex and labor‐intensive manual processes, including muti‐step reactions and intricate condition control.^[^ ^12^ ^]^

DNA exhibits the advantages of precise control due to Watson‐Crick base pairing, which allows for the design and construction of DNA structures with high accuracy.^[^ ^13^ ^]^ The synthesis of DNA sequence can be efficiently automated synthesis by a “DNA solid‐phase synthesizer”. Upon inputting the instructions, the desired DNA sequence can be automatically output with high efficacy. As such, DNA nanotechnology has emerged as a nanoscale drug delivery platform during the past decade.^[^ ^14^ ^]^ For instance, DNA nanorobot delivering thrombin was applied to induce intravascular thrombosis for tumor inhibition.^[^ ^15^ ^]^ Moreover, lysosome‐activated tetrahedral framework nucleic acids I were also utilized as a general platform for short interfering RNA (siRNA) delivery.^[^ ^16^ ^]^ Additionally, an acid‐resistant and pH‐responsive DNA hydrogel was designed for insulin delivery.^[^ ^17^ ^]^ Serving as an efficient delivery system, DNA can be engineered into a tubular nanodevice for co‐delivery of siRNA and chemo‐drug, enabling combination cancer therapy.^[^ ^18^ ^]^ Nevertheless, DNA nanostructure has not been engineered as a delivery system for STING activation yet.

**Scheme 1:**
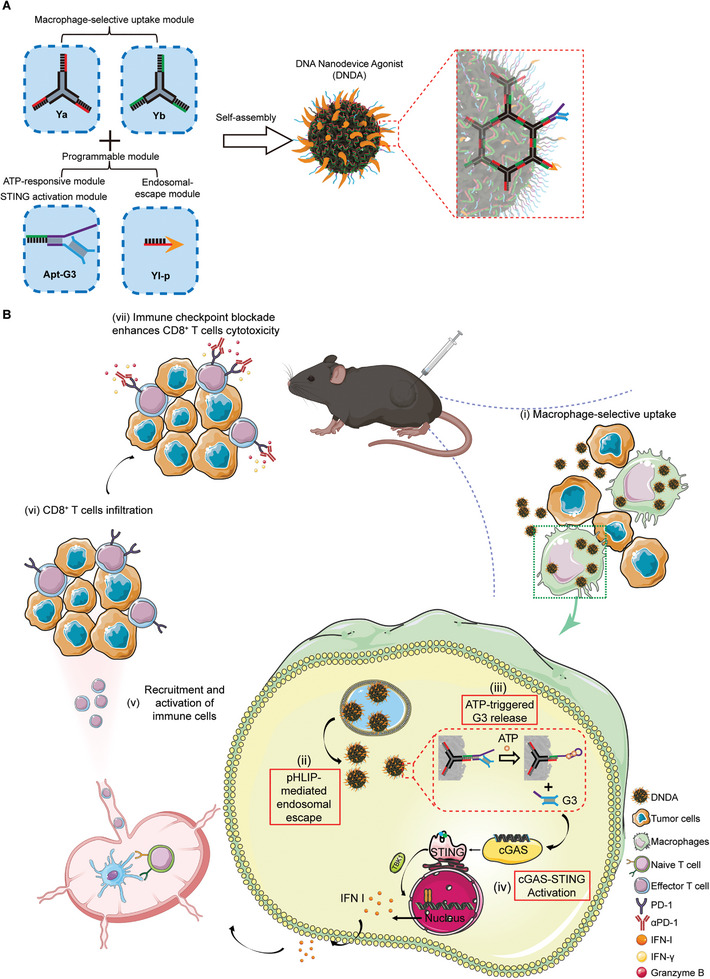
Schematic illustration of DNA nanodevice agonist (DNDA). A) Construction of DNDA based on DNA nanotechnology. B) Mechanism for cGAS‐STING activation and enhanced antitumor immunity by DNDA. After macrophage‐selective uptake due to the polypod‐like nanostructured DNA and nanoparticle size, DNDA reaches the cytosol by pHLIP‐mediated endosomal escape. In the presence of cytosolic ATP, the ATP aptamer restores its conformation to bind ATP, resulting in the dissociation of G3 and thus cGAS recognition for signaling activation.

Inspired by DNA's intrinsic property of being recognized by cGAS for cGAMP production, we conceived the idea of harnessing DNA as a carrier and agonist simultaneously. In this strategy, we engineered DNA into a DNA nanodevice agonist (DNDA) to accomplish macrophage‐selective uptake and programmable STING activation by simply self‐assembly. The DNDA consists of four functional modules: 1) a macrophage‐selective uptake module: two polypod‐like nanostructured DNAs are designed with more favorable uptake by macrophages via scavenger receptor 1 (MSR1),^[^ ^19^ ^]^ 2) an endosomal escape module: a pH‐low insertion peptide (pHLIP) enables endosomal escape for cytoplasmic release of DNDA; 3) an ATP‐responsive module: the ATP aptamer restores its conformation to bind cytosolic ATP, resulting in the dissociation of dsDNA; 4) a STING activation module: a Y‐formed short dsDNA segment G3 has been reported to potently activate cGAS‐STING.^[^ ^20^ ^]^ (**Scheme** **1**A) Through Watson‐Crick base pairing, four functionalized modules self‐assemble into a macrophage‐selective and programmable DNA nanodevice agonist. As a proof of concept, we injected DNDA intratumorally, which is consistent with the route of administration for STING agonists in clinical trials, to explore the efficacy of anti‐tumor immunotherapy. By doing so, the immunosuppressive tumor microenvironment (TME) was rebuilt with increased tumor‐infiltrated CD8^+^ T cells (Scheme 1B), and DNDA thus showed enhanced antitumor efficacy in combination with PDL1 antibody in MC38 colon cancer and B16 melanoma animal models. Our work broadens the application of DNA‐based nanotechnology, providing a new strategy for engineering a DNA nanodevice as an immune stimulator for cGAS‐STING activation.

## 2 Results and Discussion

### 2.1 Engineering the Modularized DNDA

**Figure 1:**
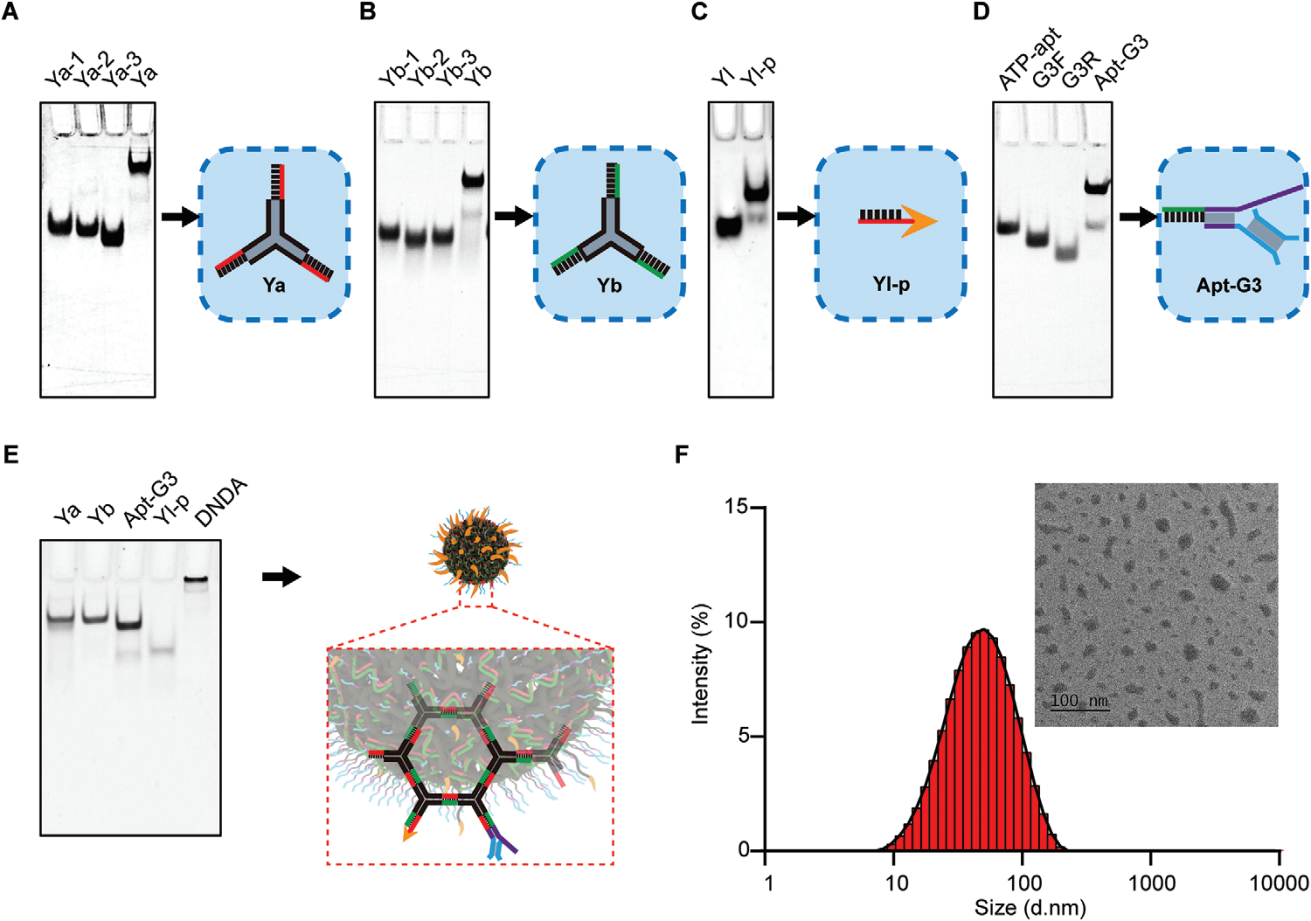
Construction of modularized DNDA. Synthesis of polypod‐like macrophage‐selective uptake modules A) Ya and B) Yb by base complementary paring. C) Synthesis of the endosomal escape module Yl‐p by conjugating a DNA linker with endosome‐releasing peptide pHLIP through a thiol‐maleimide reaction. D) Synthesis of Apt‐G3, an assembly consisting of an ATP‐responsive module (an ATP aptamer) complementary to the STING activation module (G3 segment). E) Synthesis of DNDA by self‐assembly of Ya, Yb, Yl‐p, and Apt‐G3 at a molar ratio of 1:1:1:1. F) Size and morphological characterization of DNDA using DLS and TEM. (Scale bar: 100 nm).

First, we prepared four functional DNA modules, including two polypod‐like DNA structures as the macrophage‐selective uptake module, an endosomal escape module, an ATP‐responsive module, and a STING activation module. The polypod‐like macrophage‐selective uptake modules, Ya and Yb, were both formed by complementary base pairing of three single‐stranded DNA segments (**Figure** **1**A,B). We next synthesized the endosomal escape module, Yl‐p, by modifying a DNA linker (Yl) with the endosome‐releasing peptide pHLIP through a thiol‐maleimide reaction. The synthesis of the DNA‐peptide conjugate was characterized by native polyacrylamide gel electrophoresis (PAGE) and mass spectrum (Figure 1C; Figure S1, Supporting Information). The ATP‐responsive module and the STING activation module were comprised of an ATP‐aptamer complementary with cGAS‐STING‐activating double‐stranded G3 sequence (Figure 1D). The three single‐stranded ends of Ya and Yb are all complementary to those of Apt‐G3 and Yl‐p, respectively. The as‐prepared DNA modules were then incubated at a molar ratio of 1:1:1:1 for self‐assembly into a DNA nanostructure, which was verified by native PAGE analysis (Figure 1E). DNDA is 40 nm, on average, as characterized by dynamic light scattering (DLS) and transmission electron microscope (TEM) (Figure 1F).

To confirm the in vitro safety of DNDA, we evaluated the cellular toxicity on murine macrophage‐like RAW264.7 cells. The cell viability showed no significant difference after being incubated with different concentrations of DNDA, indicating that DNDA is non‐cytotoxic in cells (Figure S2, Supporting Information). We also tested the serum stability and storability of DNDA. After co‐incubation with 10% FBS for indicating times, the particle size of DNDA showed no significant change for up to 24 h, demonstrating good serum stability (Figure S3A, Supporting Information). Similarly, no significant change in the size of DNDA was observed after lyophilization, showing good storability (Figure S3B, Supporting Information).

### 2.2 Macrophage‐Selective Uptake of DNDA

**Figure 2:**
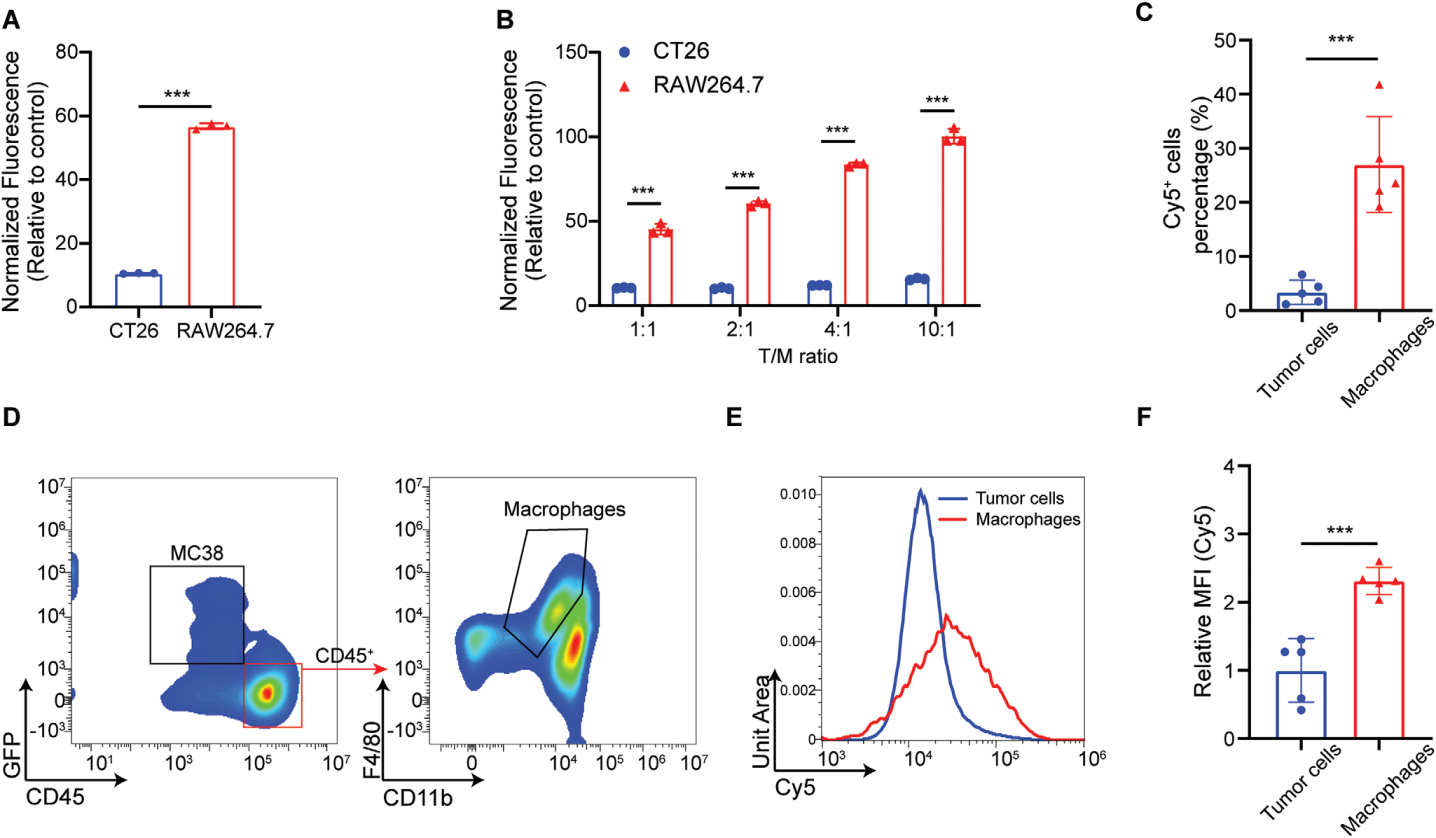
Macrophage‐selective uptake of DNDA. A) Quantitative MFI analysis of Cy5‐labeled DNDA uptake in CT26 and RAW264.7 cells. Data are shown as Mean ± SD (n = 3), and statistical significance was calculated via a two‐tailed unpaired *t*‐test, ^***^ *p* < 0.001. B) The cellular uptake level of Cy5‐labeled DNDA in CT26 cocultured with RAW264.7 in indicated tumor cell/macrophage (T/M) ratios. Data are shown as Mean ± SD (n = 3), and statistical significance was calculated via two‐way ANOVA with Sidak's post hoc test, ^***^ *p* < 0.001. C) Percentage of Cy5^+^ tumor cells (gated on L/D^−^ Cy5^+^ GFP^+^ cells) and Cy5^+^ macrophages (gated on L/D^−^ Cy5^+^ CD45^+^ CD11b^+^ F4/80^+^ cells) in DNDA‐Cy5^+^ cells after injected with Cy5‐labeled DNDA in MC38‐GFP tumor‐bearing mice. Data are shown as Mean ± SD (n = 5), and statistical significance was calculated via two‐tailed unpaired *t*‐test, ^***^ *p* < 0.001. D) Representative flow cytometric plots of Cy5^+^ GFP^+^ tumor cells and Cy5^+^ macrophages in tumors. E,F) Quantitative Cy5 MFI analysis in tumor cells and macrophages at 12 h post intratumoral injection of Cy5‐labeled DNDA. Data are shown as the Mean ± SD (n = 5), and statistical significance was calculated via a two‐tailed unpaired *t*‐test, ^***^ *p* < 0.001.

Previous study has shown that some oncogenes can antagonize cGAS‐STING activation in tumor cells, but to a far lesser degree when compared to activated macrophages when treated with the same STING agonist.^[^ ^21^ ^]^ The currently used STING agonist lacks cell‐type selectivity, leading to massive cellular uptake in tumor cells, but far less effective cGAS‐STING activation.^[^ ^7^ ^]^ Polypod‐like nanostructured DNA can be efficiently taken up by murine macrophage‐like RAW264.7 cells,^[^ ^19a^ ^]^ besides, nanoparticles of 30 to 3 µm in size are prone to macrophage uptake.^[^ ^22^ ^]^ Thus, the DNA nanodevice in our study is thought to have a higher level of cellular uptake in macrophages than that in tumor cells. To test this hypothesis, we prepared Cy5‐labeled DNDA and used flow cytometry to measure the cellular uptake level in two cell types. Results showed that the cellular uptake level of DNDA in RAW264.7 cells, a murine macrophage cell line, was 3.6‐fold over that of CT26 cells, a murine colon cancer cell line (**Figure** **2**A; Figure S4, Supporting Information). We also established a CT26/RAW264.7 co‐culture model with different Tumor cell/Macrophage (T/M) ratios to mimic the TME (Figure S5, Supporting Information, right panel), in which RAW264.7 cells were labeled with CFSE, a living cell dye. The mean fluorescent intensity of Cy5 in the two cell types was measured separately. When the T/M ratio was 1:1, 2:1, 4:1, and 10:1, the cellular uptake level of DNDA in RAW264.7 cells was 2.9, 4.0, 4.2, and 6.0 times over that of CT26 cells, respectively (Figure 2B). By our calculation, more than half of the given DNDA was phagocytosed by macrophages when the T/M ratio reached no less than 4:1. Even when macrophages were greatly outnumbered by tumor cells (T/M = 10:1), they still internalized 30% of the nanodevice (Figure S5, Supporting Information). To validate that macrophage‐selective uptake is mediated by polypod‐like nanostructure via MSR1, we designed and synthesized a linear DNDA (L‐DNDA) without a Y‐shaped backbone (Figure S6A, Supporting Information). After incubation with L‐DNDA and DNDA respectively, the uptake in RAW264.7 cells was measured. Results showed that the mean fluorescent intensity (MFI) of DNDA was 5.11 times higher than that of L‐DNDA, suggesting a more favorable macrophage uptake of polypod‐like DNA nanostructure than that of linear DNA nanostructure. Moreover, upon MSR1 inhibition by dextran sulfate,^[^ ^19^, ^23^ ^]^ the uptake of DNDA decreased by 6.76‐fold (Figure S6B, Supporting Information), validating the crucial role of modules Ya and Yb in macrophage‐selective uptake by MSR1.

To further validate the macrophage‐favored uptake in vivo, we established tumor‐bearing mice with GFP‐transfected MC38 cells, and performed intratumoral injection with Cy5‐labeled DNDA. The tumor tissues were collected at 12 h post‐injection for flow cytometry analysis. We found that the Cy5‐positive cell number of macrophages (CD11b^+^ F4/80^+^ gated on GFP^−^ CD45^+^) was 7.96 folds higher than that of tumor cells (GFP^+^ CD45^−^) (Figure 2C,D; Figure S7, Supporting Information). The uptake levels in macrophages and tumor cells were then evaluated by comparing the MFI (Figure S8, Supporting Information). The normalized MFI of macrophages was 2.31 folds over that of tumor cells (Figure 2E,F), consistent with the in vitro study. We also compared the cellular uptake of DNDA by natural killer (NK) cells and DC. The results indicated that the proportion of Cy5^+^ macrophages among Cy5^+^ cells is 8.33 times higher than that of DC and 7.93 times higher than that of NK cells, respectively (Figures S9A, S10, and S11, Supporting Information). Besides, the population of macrophages exceeds that of NK cells and DC (Figure S9B, Supporting Information). The macrophage‐selective uptake and quantitative advantage contributed to the enhanced uptake of DNDA by macrophages.

Also, the immunofluorescent imaging of the tumor tissue cryosection depicted well‐distributed DNDA that was colocalized with macrophages (Figure S12, Supporting Information). Together, these data indicated that DNDA was featured with macrophage‐selective uptake in vitro and in vivo, which may potentiate macrophage‐selective in situ STING activation and imply a more efficient way for antitumor immune stimulation.

### 2.3 Programmable cGAS‐STING Activation Process of DNDA

**Figure 3:**
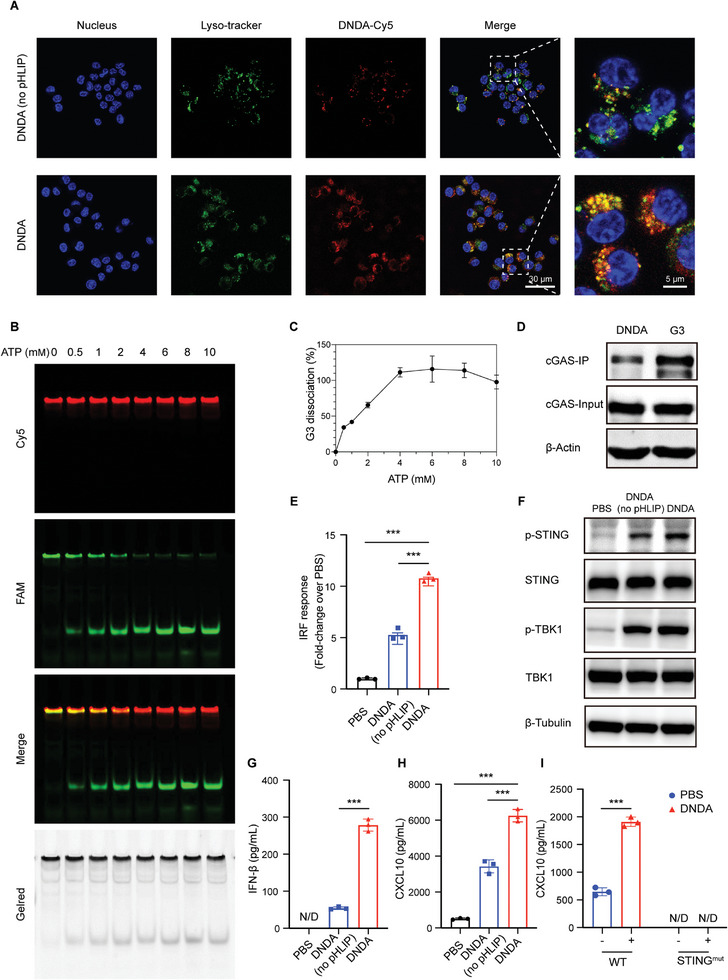
Programmable cGAS‐STING activation process of DNDA. A) Endosomal escape of Cy5‐labeled DNDA with or without pHLIP modification in RAW264.7 cells imaged by LSCM. (Scale bar: 30 µm) B) Fluorescent native PAGE analysis of dual fluorescent‐labeled DNDA treated with indicated concentration of ATP at 37 °C for 2 h. C) Quantitative grayscale analysis of G3 dissociation rate over ATP concentration. D) The binding between G3‐biotin, DNDA‐biotin, and cGAS using co‐IP analysis after co‐incubation with RAW264.7 lysate for 6 h. E) IFN regulatory factor (IRF) response elicited by DNDA with or without pHLIP in RAW ISG cells with an IRF‐inducible luciferase reporter. Data are shown as Mean ± SD (n = 3), and statistical significance was calculated via one‐way ANOVA with Tukey's post hoc test, ^***^ *p* < 0.001. F) Immunoblotting assay of phosphate STING and TBK1 level in RAW264.7 cells after incubation with DNDA with or without pHLIP. G) IFN‐β and (H) CXCL10 expression level in RAW264.7 macrophages incubated with PBS and DNDA with or without pHLIP, as measured by ELISA assay. (N/D, not detected) Data are shown as Mean ± SD (n = 3), and statistical significance was calculated via one‐way ANOVA with Tukey's post hoc test, ^***^ *p* < 0.001. I) CXCL10 expression level in DNDA‐treated BMDM cells from WT and STING^mut^ mice. (N/D, not detected) Data are shown as Mean ± SD (n = 3), and statistical significance was calculated via two‐way ANOVA with Sidak's post hoc test, ^***^ *p* < 0.001.

An intracellular barrier for cGAS‐STING agonist, as previously noted, involves endosomal escape to the cytosol where cGAS‐STING finally locates, thus preventing its lysosomal degradation.^[^ ^8^, ^24^ ^]^ In our study, we used the endosome‐releasing peptide pHLIP, which has been reported to mediate acid‐activated membrane insertion and endosomal escape for such purpose.^[^ ^25^ ^]^ We herein confirmed DNDA's capability for endosomal escape in RAW264.7 cells by using laser scanning confocal microscopy (LSCM), as compared to its counterpart without pHLIP modification (**Figure** **3**A). Co‐localization analysis using Manders’ co‐localization coefficient (MCC) showed that DNDA co‐localizes with lysosomes to a significantly lesser extent than DNDA (no pHLIP) (Figure S13, Supporting Information). Further, the line scan profiles illustrated that the fluorescence intensities of DNDA‐Cy5 and lysosomes exhibit substantial overlap in DNDA (no pHLIP) group. While in DNDA group, the intensities of two fluorescent signals are not concurrent (Figure S14, Supporting Information). The co‐localization analysis collectively indicated that, with the assistance of pHLIP, DNDA successfully escaped from the lysosome and entered the cytoplasm.

Another intracellular barrier for cytosolic cGAS‐STING agonism is the dissociation of the agonist from the delivery carrier to diminish steric hindrance and allow cGAS recognition. We thus incorporated an ATP aptamer for cytosolic ATP‐triggered G3 release. To test the releasing profile in response to ATP, we prepared dual fluorescent‐labeled DNDA with Cy5 on one of the backbone structures (Ya‐Cy5) and FAM on the activating module (Apt‐G3‐FAM). The release of G3 could thus be detected through fluorescent PAGE gel imaging. Without the presence of ATP, FAM showed colocalization with the Cy5 signal, representing the intact DNDA nanostructure. When followed by the addition of ATP, however, a gradually increasing signal of the FAM‐labeled G3 band was observed (Figure 3B). Quantitative analysis showed complete G3 release from the nanostructure in the presence of ATP over 2 mM (Figure 3C), indicating a good releasing profile in response to the intracellular ATP level up to 10 mm. After the dissociation of the activating module G3, its recognition by cGAS is the prerequisite for cGAS‐STING activation. The binding between DNDA and cGAS was confirmed in our study by co‐immunoprecipitation (Co‐IP) analysis using biotin‐labeled G3 to bind to streptavidin beads and to pull down the cytosolic cGAS, which showed a dynamic binding and dissociating process (Figure S15, Supporting Information). Further, the DNDA incorporating biotin‐G3 also showed strong binding to the intracellular cGAS at 6 h post‐incubation with RAW264.7 cell lysate (Figure 3D). Taken together, DNDA showed great ability to escape from the endosome, release activating module, and bind to cGAS, which are the basis for cGAS‐STING activation.

Based on the well‐clarified intracellular fate of DNDA, we next evaluated the cGAS‐STING activating ability of DNDA using RAW264.7 cells stably expressing an interferon regulatory factor (IRF)‐inducible luciferase reporter (RAW ISG). DNDA showed robust cGAS‐STING activation over PBS control (Figure 3E). We also found that DNDA, with or without pHLIP modification, has comparable cellular uptake levels in macrophages (Figure S16, Supporting Information), indicating that the difference between their activating ability is not dependent on cellular uptake. We also observed the elevated phosphorylation level of STING and its downstream TANK binding kinase 1 (TBK1) by immunoblotting (Figure 3F). The activation of cGAS‐STING was also confirmed by detecting the elevated expression of STING‐related cytokines in RAW264.7 cells in both mRNA (Figure S17, Supporting Information) and protein levels (Figure 3G,H). The DNA nanodevice without pHLIP modification also showed unexpected mild cGAS‐STING activation in vitro (Figure 3E–H), presumably because DNA nanostructures could enter cells via both endocytosis and endocytosis‐independent mechanisms.^[^ ^26^ ^]^ Nevertheless, DNDA apparently elicited more efficient endosomal escape and more robust cGAS‐STING agonism, compared to its counterpart without pHLIP modification.

Next, we prepared bone marrow‐derived macrophage (BMDM) from wide‐type (WT) mice and STING^mut^ mice. Increased expression of CXCL10 upon DNDA treatment was found in BMDM from WT mice, but not from STING^mut^ mice, demonstrating the robust cGAS‐STING activation (Figure 3I).

### 2.4 cGAS‐STING Activation by DNDA in Tumor‐Bearing Mice

**Figure 4:**
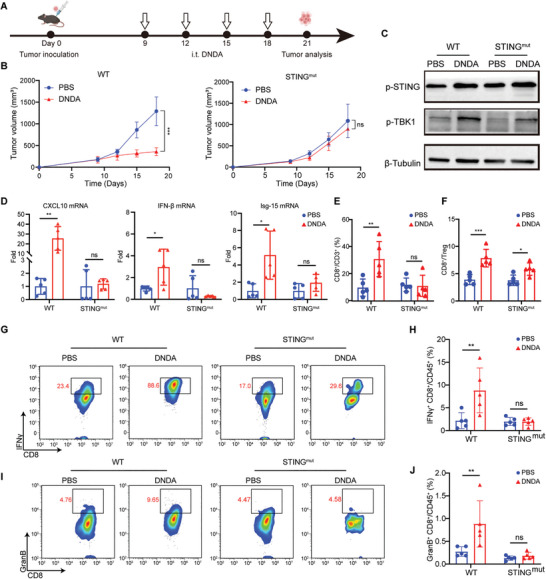
cGAS‐STING activation by DNDA in tumor‐bearing mice. A) Treatment schedule of DNDA in MC38 tumor‐bearing C57BL/6 WT and STING^mut^ mice. B) Tumor growth curve of MC38 tumors with indicated treatment in C57BL/6 WT mice and STING^mut^ mice. Data are shown as Mean ± SD (n = 5), statistical significance was calculated via two‐way ANOVA with Tukey's post hoc test, ^***^ *p* < 0.001, ns means no significance. C) Immunoblotting assay of STING and TBK‐1 phosphorylation level and D) Real‐Time PCR analysis of mRNA level of CXCL10, IFN‐β, and Isg‐15 in tumor tissues from WT or STING^mut^ mice with indicated treatment. Data are shown as Mean ± SD (n = 5), statistical significance was calculated via two‐way ANOVA with Sidak's post hoc test, ^*^ *p* < 0.05, ^**^ *p* < 0.01, ns means no significance. Quantitative analysis of E) tumor‐infiltrated CD8^+^ T cells (gated on L/D^−^ CD3^+^ CD8^+^ cells) and F) the ratio between tumor‐infiltrated CD8^+^ T cells (gated on L/D^−^ CD3^+^ CD8^+^ cells) and regulatory T cells (gated on L/D^−^ CD3^+^ CD4^+^ Foxp3^+^ cells). Data are shown as Mean ± SD (n = 5), statistical significance was calculated via two‐way ANOVA with Sidak's post hoc test, ^*^ *p* < 0.05, ^**^ *p* < 0.01, ^***^ *p* < 0.001, ns means no significance. Representative flow cytometric plots of G) IFNγ^+^ CD8^+^ T cells and I) GranB^+^ CD8^+^ T cells in tumors on day 21 post‐tumor inoculation. The frequency of H) IFNγ^+^ CD8^+^ T cells (gated on L/D^−^ CD45^+^ CD3^+^ CD8^+^ IFNγ^+^ cells) and J) GranB^+^ CD8^+^ T cells (gated on L/D^−^ CD45^+^ CD3^+^ CD8^+^ Granzyme B^+^ cells) in tumors analyzed on day 21 post tumor inoculation. Data are shown as the Mean ± SD (n = 5), statistical significance was calculated via two‐way ANOVA with Sidak's post hoc test, ^**^ *p* < 0.01, ns means no significance.

To evaluate the in vivo activation of DNDA, we established MC38 tumor‐bearing C57BL/6 WT and STING^mut^ mice. Intratumoral injection of DNDA was conducted four times with a three‐day interval starting from day 9 post‐tumor inoculation, and the tumor volume was measured (**Figure** **4**A). Upon DNDA treatment, the tumor growth rate was greatly inhibited in WT mice, while almost no inhibition was observed in STING^mut^ mice (Figure 4B; Figure S18, Supporting Information). We then randomly selected three tumor samples in each group for immunoblotting analysis and found that DNDA could effectively activate the cGAS‐STING signal transduction in WT mice by phosphorylation of TBK1 and STING. (Figure 4C; Figure S19, Supporting Information). Furthermore, the mRNA levels of STING‐relevant cytokines, CXCL‐10, IFN‐β, and Isg15, were remarkably elevated in DNDA‐treated tumor tissues in WT mice, whereas no significant differences were found in STING^mut^ mice (Figure 4D). Next, the tumor tissues were collected for flow cytometry analysis. It was found that the tumor‐infiltrated CD8^+^ T cells, as well as the ratio of CD8^+^ T cells to Treg cells, were increased by DNDA in WT mice (Figure 4E,F; Figure S20, Supporting Information). Also, the IFNγ^+^ CD8^+^ and GranB^+^ CD8^+^ effector T cells were found to be increased in DNDA‐treated WT mice by 4.05‐fold and 3.17‐fold, respectively, compared to that in the PBS group (Figure 4G–J; Figure S21, Supporting Information). Consistent with previous report,^[^ ^27^ ^]^ CD4^+^ T cells were also increased upon cGAS‐STING activation (Figure S20A, Supporting Information). This is mainly because STING agonists can induce the production of pro‐inflammatory cytokines, such as type I IFNs, TNF‐α, and CXCL10, thus promoting CD4^+^ T cell differentiation.^[^ ^28^ ^]^ Furthermore, CD4^+^ T cells are considered to play a crucial role in promoting CD8^+^ T cell recruitment and activation,^[^ ^29^ ^]^ resulting in strong anti‐tumor immune response

Taken together, the above results indicated that DNDA could promote the cGAS‐STING signaling transduction for enhanced T‐cell infiltration and activation in the tumor microenvironment.

### 2.5 Therapeutic Effects of DNDA on Inhibiting Tumor Growth Combined with PDL1 Blockade

**Figure 5:**
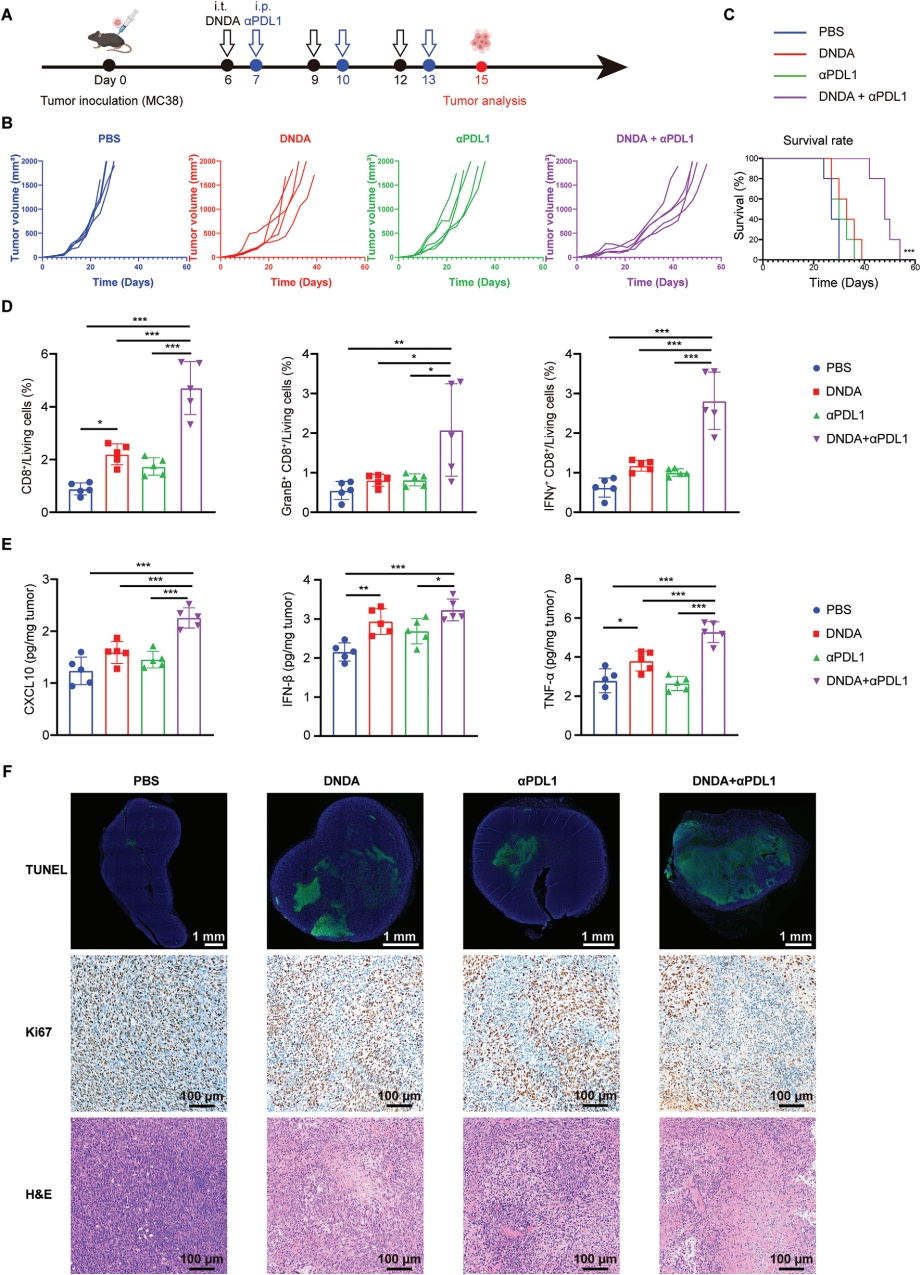
Therapeutic effects of DNDA on inhibiting tumor growth combined with PDL1 blockade in MC38 tumor‐bearing mice. A) Treatment schedule of DNDA and anti‐PDL1 combination therapy on MC38 bearing C57BL/6 mice. B) Individual tumor growth curve and C) overall survival rate of MC38 bearing C57BL/6 mice with indicated treatment (n = 5). Statistical significance was calculated via the log‐rank Mantel‐Cox test, ^***^ *p* < 0.001. D) Quantitative analysis of tumor‐infiltrated CD8^+^ T cells (gated on L/D^−^ CD3^+^ CD8^+^ cells), IFNγ^+^ CD8^+^ T cells (gated on L/D^−^ CD45^+^ CD3^+^ CD8^+^ IFNγ^+^ cells) and GranB^+^ CD8^+^ T cells (gated on L/D^−^ CD45^+^ CD3^+^ CD8^+^ Granzyme B^+^ cells). Data are shown as Mean ± SD (n = 5), and statistical significance was calculated via one‐way ANOVA with Tukey's post hoc test, ^*^ *p* < 0. 05, ^**^ *p* < 0.01, ^***^ *p* < 0.001. E) ELISA analysis of CXCL10, IFN‐β, and TNF‐α excretion in tumor tissues. Data are shown as Mean ± SD (n = 5), statistical significance was calculated via one‐way ANOVA with Tukey's post hoc test, ^*^ *p* < 0. 05, ^**^ *p* < 0.01, ^***^ *p* < 0.001. (F) TUNEL, Ki67, and H&E staining of tumor tissue sections from mice with indicated treatment.

In clinic, only a small subset of patients responds well to ICB treatment, due to the immunosuppressive TME and lack of immune cell infiltration. As the bridge between innate immunity and adaptive immunity, cGAS‐STING agonists are believed to potentiate ICB therapy. In our study, we have also demonstrated that DNDA was capable of in situ cGAS‐STING activation and T‐cell recruitment. We thus hypothesized it is a rational solution to improve the PDL1 blockade therapy in combination with STING‐activating nanodevice treatment. To test this hypothesis, MC38 tumor‐bearing mice were treated with DNDA by intratumoral injection on day 6 post‐tumor inoculation, followed by systemic anti‐PDL1 antibody (αPDL1) administration on the next day for 3 cycles. (Figure S22A, Supporting Information). The combinational group showed better efficacy in suppressing tumor growth than monotherapy (Figures S22 and S23, Supporting Information). Also, the combination therapy significantly prolonged the survival compared to either DNDA or αPDL1 treatment (**Figure** **5**B,C). Then median survival upon treatment by PBS,

DNDA, αPDL1, and combination therapy were 27 days, 33 days, 30 days, and 48 days, respectively. To further explore the antitumor mechanism, we performed a flow cytometry analysis of tumor tissues two days post‐treatment to investigate the infiltration and activation of CD8^+^ T cells. Our results showed a 5.3‐, 2.1‐ and 2.7‐fold increase in CD8^+^ T cells by combination therapy, compared to PBS, DNDA, and αPDL1 monotherapy, respectively. An elevated level of IFNγ^+^ and GranB^+^ CD8^+^ T cells was also detected (Figure 5D; Figure S24, Supporting Information). Next, we tested the secretion of STING‐related cytokines and found out that DNDA+αPDL1 group showed increased CXCL10 secretion both in tumors and plasma. Moreover, the combination therapy elicited the secretion of IFN‐β and TNF‐α in tumors and plasma, further validating the activation of an antitumor immune response, which was aligned with the activation of tumor‐specific T cells (Figure 5E; Figure S25, Supporting Information). The therapeutic effect was further confirmed by TUNEL staining and H&E staining (Figure 5F), which showed obvious apoptosis in tumor tissues in DNDA+αPDL1 group. Immunohistochemical staining of tumor sections confirmed that DNDA+αPDL1 treatment downregulated Ki67 expression (Figure 5F), indicating the inhibition of tumor cell proliferation. Taken together, these results provided convincing evidence that STING‐activating DNDA can prime the immunosuppressive TME for enhanced infiltration of effective T cells, and enhanced antitumor immunity by combined ICB treatment.

**Figure 6:**
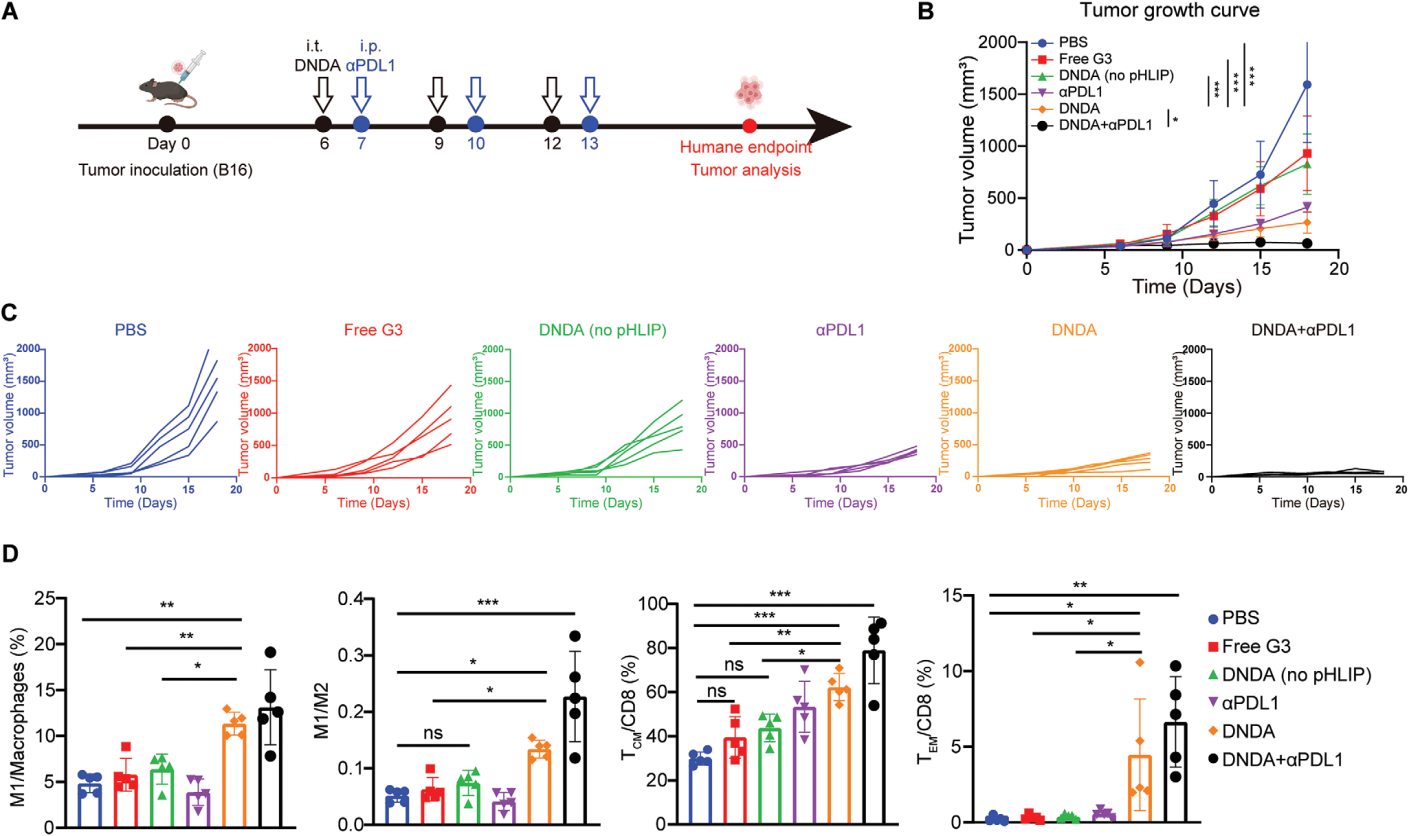
Therapeutic effects of DNDA on inhibiting tumor growth combined with PDL1 blockade in B16 tumor‐bearing C57BL/6 mice. A) Treatment schedule of DNDA and anti‐PDL1 combination therapy. B) Tumor growth curve and C) individual tumor growth curve with indicated treatment. Data are shown as Mean ± SD (n = 5), and statistical significance was calculated via two‐way ANOVA with Tukey's post hoc test, ^*^ *p* < 0. 05, ^***^ *p* < 0.001. D) Quantitative analysis M1 macrophages (gated on L/D^−^ CD45^+^ F4/80^+^ CD11b^+^ CD206^−^ CD86^+^), the ratio of M1 to M2 macrophages (gated on L/D^−^ CD45^+^ F4/80^+^ CD11b^+^ CD206^+^) in tumor, T_CM_ (gated on L/D^−^ CD45^+^ CD3^+^ CD8^+^ CD62L^+^ CD44^+^) and T_EM_ (gated on L/D^−^ CD45^+^ CD3^+^ CD8^+^ CD62L^−^ CD44^+^) cells in tumors. Data are shown as Mean ± SD (n = 5), statistical significance was calculated via one‐way ANOVA with Tukey's post hoc test, ^*^ *p* < 0. 05, ^**^ *p* < 0.01, ^***^ *p* < 0.001, ns means no significance.

To further validate the therapeutic effect of DNDA combined with ICB, the antitumor efficacy was tested in another tumor model. B16 melanoma tumor‐bearing mice were treated with DNDA by intratumoral injection 6 days after tumor inoculation, followed by systemic anti‐PDL1 antibody (αPDL1) administration on the next day for 3 cycles (**Figure** **6**A). Tumor volume was measured every 3 days to monitor the tumor growth until 1D of the tumor reached 20 mm (Figure 6B,C). The combination therapy suppressed tumor growth compared with DNDA or αPDL1 alone. Of note, tumor growth was significantly suppressed by DNDA, compared with free G3 and DNDA (no pHLIP). The tumor growth inhibition (TGI) in the DNDA group reached 79.71%, which is 2.76 times higher than that of free G3 (28.88%) and 1.44 times higher than that of DNDA (no pHLIP) (55.18%), confirming the rational design of programmable activation of cGAS‐STING (Figure 6A,B; Figure S26, Supporting Information). To study the tumor microenvironment after DNDA and combination therapy, we investigated macrophage polarization and T memory cells by flow cytometry. The DNDA‐treated group showed an increase in the number of M1 macrophages. In addition, the ratio of M1 to M2 macrophages was increased by 2.60, 2.13, and 1.81 times compared to the PBS group, free G3 group, and DNDA (no pHLIP) group, respectively (Figure 6D; Figure S27, Supporting Information). These results demonstrated that DNDA promoted the polarization of macrophages toward the pro‐inflammatory M1 phenotype. Notably, the percentage of central memory T cells (T_CM_) and effector T cells (T_EM_) were elevated in the combination therapy group, suggesting the reverse of the immunosuppressive tumor microenvironment (Figure 6E; Figure S28, Supporting Information).

To explore the mechanism of T cell infiltration and activation in tumors, we investigated the maturation of DC in tumor‐draining lymph nodes (tdLN). Upon DNDA administration, the density of mature DC upregulated 2.34‐fold compared with the PBS group (Figure S29, Supporting Information). These results confirmed that the cGAS‐STING activation by DNDA can promote the maturation of DC in tdLN.

The body weights were measured every 3 days and showed no difference between PBS and DNDA groups (Figure S30A, Supporting Information). Intratumoral administration of DNDA did not cause changes in blood chemistry (Figure S30B, Supporting Information) and hematologic parameters (Table S2, Supporting Information). The H&E staining did not show significant toxicity in major organs (Figure S30C, Supporting Information). These results suggested that DNDA had good biocompatibility with intratumoral administration.

The effective clinical response of ICB is dependent on the tumor immune microenvironment by restoring the adaptive immune system for combating tumors. Our strategy of using the DNA nanodevice to activate cGAS‐STING has been demonstrated to effectively act as a bridge between innate and adaptive immune for improved antitumor immunotherapy.

The design of STING agonists and delivery carriers play a crucial role in the activation of cGAS‐STING pathway. Previous studies have typically used cyclic dinucleotides (CDNs) and non‐nucleotide compounds as agonists to directly recognize STING for its activation,^[^ ^30^ ^]^ and meanwhile employed polymer‐ or lipid‐based systems as delivery carriers. In this study, inspired by cGAS being a natural DNA sensor, we employed DNAs both as an agonist module and a delivery carrier module to achieve cGAS‐STING activation. Our DNA‐modularized strategy possesses two major features.

First, STING exhibits species differences between rodents and humans.^[^ ^31^ ^]^ In particular, human STING has five haplotypes that have different sensitivity toward the small‐molecular agonists.^[^ ^32^ ^]^ These differences pose a challenge in the design and screening of STING agonists. Our strategy of using a specific DNA structure to target upstream cGAS may circumvent this challenge since the DNA recognition pattern of cGAS is consistent among different species.

Second, when delivering STING agonists using polymer‐based systems, it is necessary to integrate multiple functionalities into the delivery vehicle. This process involves various chemical modifications and multi‐step synthesis, making it a complex process. In contrast, by taking advantage of DNA's precise base pairing, we have applied DNA as a delivery vehicle to make the assembly of functional modules more controllable. Through the modular design of DNA, this approach can achieve macrophage‐selective uptake, lysosomal escape, and ATP response in a programmable manner.

## 3 Conclusion

In conclusion, we have developed a DNA nanodevice agonist for cGAS‐STING activation. Without time‐consuming screening for either multi‐functional delivery materials or developing new small molecular agonists, we provide a new strategy of using a smart DNA nanostructure for boosting innate immune response. DNDA can specifically target and activate TAMs by its polypod‐like nanostructure and appropriate nanoparticle size. By activating cGAS‐STING in TAMs, the immunosuppressive TME was converted with the increased level of proinflammatory cytokines and chemokines, as well as enhanced tumor infiltration of cytotoxic T cells. With the ameliorated tumor immune TME, the combined therapy with DNDA and ICB has shown a synergistic therapeutic effect in inhibiting tumor growth. Our study has broadened the application of DNA nanotechnology into the immune regulator for cell‐selective agonism, and paved the way for exploiting such therapeutic strategy for enhanced antitumor immunity. Additionally, our work has also enlightened a versatile platform for using such DNA nanodevice for the cell‐specific delivery of other nucleotide drugs, such as mRNA and siRNA, in a biocompatible manner.

## 4 Experimental Section

### Materials

Chemicals and reagents were purchased from Sigma‐Aldrich (USA) unless stated otherwise. All oligonucleotides (see Table S1, Supporting Information) and pH‐low insertion peptide (pHLIP, sequence: ACDDQNPWRAYLDLLFPTDTLLLDLLW)^[^ ^33^ ^]^ used for preparing DNDA in this study were synthesized and purified by Sangon Biotech Co. (Shanghai, China). Each oligonucleotide was made into a 100 µm stock solution using sterilized deionized water and stored at −20 °C prior to use. Murine macrophage cell line RAW264.7 and murine colon cancer cell line CT26 were preserved in the laboratory. RAW264.7 cells transfected with an IFN response factor (IRF)‐activating luciferase reporter (RAW ISG) and luciferase detection reagent, QUANTI‐Luc, were purchased from Invivogen (USA). DNA loading buffer, Hoechst 33 342, LysoTracker® Green, Carboxyfluorescein diacetate succinimidyl ester (CFSE), ATP disodium salt, and BCA protein quantitative assay kit were purchased from Beyotime Biotechnology (Shanghai, China). GelRed® was purchased from Biotium (USA). Fetal bovine serum (FBS), trypsin‐EDTA, high glucose Dulbecco's Modified Eagle's Medium (DMEM), Roswell Park Memorial Institute (RMPI)−1640 medium, Iscove's Modified Dulbecco's Medium (IMDM) and penicillin‐streptomycin (PS) were all obtained from Gibco, Thermo Fisher Scientific (USA). STING rabbit antibody (Cat: 13647), phosphor‐STING rabbit antibody (Cat: 72971), TBK1 rabbit antibody (Cat: 38066), phosphor‐TBK1 rabbit antibody (Cat: 5483), β‐tubulin rabbit antibody (Cat: 2128) and cGAS rabbit antibody (Cat: 31659) were all purchased from Cell Signaling Technology (USA). HRP‐linked goat anti‐rabbit secondary antibody was purchased from Santa Cruz (USA). RIPA cell lysis buffer, protease/phosphatase inhibitor cocktail, and ECL substrate solution were obtained from Pierce, Thermo Fisher Scientific (USA). Mouse macrophage colony‐stimulating factor (M‐CSF) was purchased from PeproTech (USA). FITC‐linked CD11b antibody and PE‐linked F4/80 antibody were purchased from BioLegend (USA). ELISA kits for IFN‐β and CXCL10 detection were purchased from Novus Biologicals (USA). Pierce^TM^ IP lysis buffer (Cat: 87787) was purchased from Thermo Fisher Scientific (USA). Streptavidin Magnetic Beads (Cat: 65001) were purchased from Thermo Fisher Scientific (USA). RNA‐Quick Purification Kit (Cat: RN001), Tissue RNA purification Kit (Cat: RN002), Fast Reverse Transcription kit (Cat: RT001), and 2× Super SYBR Green qPCR Master Mix (Cat: QP002) were purchased from Yishan Biotech (China). Collagenase I (Cat: 40507ES60) was purchased from Yeasen (China). Dnase I (Cat: 10104159001) was purchased from Roche (Switzerland). Anti‐FcR/Purified anti‐mouse CD16/32 antibody (Cat: 101301, Clone: 93) was purchased from Biolegend (USA). Zombie NIR^TM^ Fixable Viability Kit (Cat: 423105) was purchased from Biolegend (USA). DAPI (Cat: D9542) was purchased from Sigma (USA). Anti‐mouse PDL1 (B7‐H1) (Cat: BE0101, Clone: 10F.9G2) was purchased from Bio X Cell (USA). Sulfate Dextran Sodium (CAS: 9011‐18‐1) was purchased from Bidepharm (China).

### Synthesis of DNDA—Synthesis of DNA‐peptide conjugate

Maleimide‐modified Ylinker (Yl‐mal) was dissolved in sterilized phosphate‐buffered saline (PBS) to make a 100 µm solution, followed by adding pHLIP peptide with a molar ratio of 1:5. The mixture was vortexed and reacted at room temperature for 2 h. Free DNA and peptide were removed by Zeba spinning desalting column (Thermo Fisher Scientific, USA). The Mass Spectrum of unmodified Yl and Yl‐p conjugates was carried out by Sangon Biotech Co. (Shanghai, China).

### Synthesis of DNDA—Preparation of DNA building blocks. Single‐stranded oligonucleotides

Ya‐1, Ya‐2, and Ya‐3 (100 µm) were mixed with TM buffer (20 mm Tris‐HCl, 50 mm MgCl_2_, pH 8.0) at a final concentration of 20 µm. The mixture was heated at 90 °C for 5 min and then slowly cooled to 4 °C over 4 h to prepare annealed Y‐shaped backbone structure Ya. For Cy5‐labeled Ya (Ya‐Cy5), Ya‐1 was replaced by Ya‐1‐Cy5. Another Y‐shaped backbone structure, Yb, was prepared by the same method, except that the oligonucleotides were replaced by Yb‐1, Yb‐2, and Yb‐3. The activating building block Apt‐G3, comprised of ATP‐aptamer (ATP apt), G3F, and G3R, was also prepared using the same method as that for Ya and Yb. For FAM‐labeled Apt‐G3 (Apt‐G3‐FAM), G3R was replaced by G3R‐FAM.

### Synthesis of DNDA—Preparation of DNDA nanostructure

The DNDA nanostructure was prepared by mixing Ya, Yb, G3‐apt, and Yl‐p at a molar ratio of 1:1:1:1 with TM buffer to make the final concentration of 2 µm. For DNDA without pHLIP modification, Yl‐p was replaced by unmodified Yl. The mixture was incubated at 45 °C for 10 min and 37 °C for 2 h. For fluorescent‐labeled nanostructure Ya and/or Apt‐G3 was replaced by Ya‐Cy5 and/or Apt‐G3‐FAM. The as‐prepared DNDA nanostructures were dialyzed over PBS to remove unbound oligonucleotides.

### Synthesis of DNDA—Preparation of linear DNDA (L‐DNDA)

Ya‐1 (100 µm) and Apt‐G3 (20 µm), Yc (100 µm), and Yl‐p (50 µm) were mixed with TM buffer at a molar ratio of 1:1 to make the final concentration of 10 µm, respectively. The mixture was incubated at 37 °C for 2 h to obtain Ya‐apt and Yc‐Ylp. Then Ya‐apt and Yc‐Ylp were mixed at a molar ratio of 1:1, and the mixture was incubated at 37 °C for 2 h to obtain L‐DNDA.

### Polyacrylamide Gel Electrophoretic Analysis

Samples were diluted into 0.5 µm, mixed with DNA loading buffer, and then loaded onto a 10% native PAGE gel in TAE (Tris‐acetate‐EDTA) buffer, which was run at 10 V cm^−1^ for 0.5 h. The PAGE gels were stained with 0.03% GelRed® for 10 min, followed by imaging using the Amersham Imager 680R (USA) under UV light.

### Dynamic Light Scattering Measurements

Samples (45 µL) of the final DNDA solution were dissolved in double‐distilled water (1 mL) and measured at room temperature using the Malvern Zetasizer Nano ZS (Malvern Instruments, Ltd., Worcestershire, UK). Values are reported as the mean values with standard deviation.

### Cell Culture

RAW264.7 and RAW ISG cells were cultivated in high‐glucose DMEM supplemented with 10% FBS, 100 U mL^−1^ penicillin, and 100 µg mL^−1^ streptomycin. Cells were scraped from the bottom of the 10 mm dish when reaching 80–90% confluency. Separately, CT26 cells were cultivated in RMPI‐1640 medium supplemented with 10% FBS, 100 U/mL penicillin, and 100 µg mL^−1^ streptomycin. Cells were passaged by treatment with 0.25 Trypsin‐EDTA at 37 °C for 3 min when reaching 80–90% confluency. All cells were tested for Mycoplasma contamination and cultured in a 37 °C incubator (Thermo Fisher Scientific, USA) within a humidified atmosphere of 5% CO_2_.

### In Vitro Toxicity, Serum Stability, and Storability of DNDA

RAW264.7 cells were seeded into a 96‐well plate at a density of 1×10^4^ cells per well and incubated with 0, 0.1, 0.2, 0.4, 0.4, and 0.8 µm DNDA for 24 h. After incubation, the cell viability was measured by CCK‐8 at OD450 using a plate reader (BioTek Synergy H1, USA). The serum stability of DNDA was conducted in 10% FBS at 37 °C. The size of DNDA was measured using DLS at 1, 2, 4, 8, 12, 24 h post co‐incubation. DNDA was lyophilized and reconstituted to test the storability. During the process, 5% sucrose solution was used as a cryoprotectant.^[^ ^34^ ^]^ The particle size of DNDA was compared before and after lyophilization.

### Endosomal Escape Evaluation

RAW264.7 cells were seeded into a 3.5 mm glass‐bottom dish at a density of 2×10^5^ cells per dish, followed by incubation at 37 °C for 24 h. Cells were treated with Cy5‐labeled DNDA with or without pHLIP modification for 4 h and then stained with Hoechst 33 342 and LysoTracker® Green for 30 min before imaging by laser scanning confocal microscopy (LSCM, Leica TCS SP8, Germany).

The co‐localization analysis of DNDA‐Cy5 and lysosomes was performed using ImageJ. Three individual cells were randomly selected from the field of view for analysis. Manders’ co‐localization coefficient (MCC)^[^ ^35^ ^]^ using automatic Costes thresholding was calculated for individual cells. MCC yields the fraction of the Cy5 signal that overlaps with the signal of lysosomes in the total Cy5 signal.

### ATP‐Responsive G3 Release

The dual fluorescent‐labeled DNDA nanostructure was incubated with ATP solution at a concentration of 0, 0.5, 1, 2, 4, 6, 8, and 10 mm at 37 °C for 2 h. All samples were diluted to 0.5 µm and mixed with DNA loading buffer, followed by loading onto a 10% native PAGE gel, which was further run in TAE buffer at 10 V cm^−1^ for 0.5 h. The gel was then imaged with the Amersham Imager 680R fluorescent gel imager (USA) in the FITC and Cy5 channels. Quantitative grayscale analysis of released G3‐FAM bands was further performed by ImageJ software.

### Co‐Immunoprecipitation (Co‐IP)

RAW264.7 cells were lysed with Pierce^TM^ IP lysis buffer (Thermo Fisher Scientific) mixed with protease and phosphatase inhibitor cocktail (Thermo Fisher Scientific). DNDA and G3 were synthesized by biotin‐modified G3R. After incubation with Streptavidin Magnetic Beads (Thermo Fisher Scientific) at 4 °C overnight, biotin‐modified G3 or DNDA was incubated with cell lysis for the indicated time, followed by immunoblotting assay.

### IRF Response Measurement

RAW ISG cells were seeded into a 24‐well plate at a density of 5×10^4^ cells per well, followed by incubation at 37 °C for 24 h. Cells were treated with PBS and 40 µg mL^−1^ DNDA with or without pHLIP modification for another 24 h. The cell medium of each well was collected and centrifuged to remove cell pellets. 10 µL of each sample were moved into a 96‐well white‐bottom plate, and 50 µL QUANTI‐Luc were added to each well. The luciferase activity was measured immediately by using a plate reader (BioTek Synergy H1, USA) at room temperature. For dose‐dependent IRF response, cells were treated with 20, 40, and 80 µg mL^−1^ DNDA for 24 h and detected by QUANTI‐Luc. For time‐dependent IRF response, cells were treated with 40 µg mL^−1^ DNDA for 4, 6, 8, 10, 12, and 24 h, followed by luciferase activity detection.

### Immunoblotting—In vitro analysis

RAW264.7 cells were seeded into a 24‐well plate at a density of 5×10^4^ cells per well, followed by incubation at 37 °C for 24 h. Cells were treated with PBS and 40 µg mL^−1^ DNDA with or without pHLIP modification for another 24 h. Cell medium was removed, and cells were lysed using RIPA lysis buffer mixed with protease and phosphatase inhibitors. Cell lysates were centrifuged to remove cell pellet, adjusted to the same protein concentration by BCA assay, and loaded to 10% SDS‐PAGE electrophoresis. The electroblotted PVDF membranes (Merck Millipore, USA) were incubated with STING, phosphor‐STING, TBK1, phosphor‐TBK1, β‐tubulin rabbit antibody, and HRP‐linked goat anti‐rabbit secondary antibody. The membrane was incubated with ECL substrate solution and then subjected to a gel‐imaging system (Odyssey, USA).

### Immunoblotting—In vivo analysis

Tumors were lysed with radioimmunoprecipitation assay (RIPA) buffer (Beyotime biotech) supplemented with protease/phosphatase inhibition cocktails (Thermo Fisher Scientific). After tumor tissue homogenate was completed, the lysates were incubated on ice for 5 min, and then centrifuged at 12,000 rpm for 20 min at 4 °C. Then supernatant was collected for analysis.

### Bone Marrow‐Derived Macrophage Induction

Male C57BL/6 mice were obtained from the Laboratory Animal Care Facility of Shanghai Jiao Tong University School of Medicine. STING mutant (STING^mut^ T149A) male C57BL/6 mice were a kind gift from Prof. Liufu Deng. The animal experiment designed in this study was approved by the Ethics Committee of Shanghai Jiao Tong University School of Medicine (SJTU‐SM). Animals were kept in the SJTU Animal Resource Center and given free access to food and water throughout the study. Bone marrow‐derived macrophages (BMDMs) from wide‐type (WT) or STING^mut^ mice were isolated and induced according to a previous study.^[^ ^36^ ^]^ Briefly, animals were humanely sacrificed and sterilized in 95% ethanol. The femur and tibia bones were isolated to rinse off hair and cut open. Bone marrow cells were then flushed out into cold PBS with 2% FBS using a 21G needle and 10 mL syringe. Cells were passed through a 70 µm cell strainer and incubated with 0.8% NH_4_Cl solution on ice for 10 min to remove red blood cells. Cells were then spun down at 2000 rpm for 5 min at 4 °C and resuspended in IMDM with 20 ng mL^−1^ M‐CSF. Cells were seeded into a 6‐well plate at a density of 4×10^6^ cells per well and changed to fresh medium on day 3. On day 7, the formation of mature BMDM was evaluated using flow cytometry analysis to detect cells expressing CD11b and F4/80, two macrophage surface markers.

### ELISA—In vitro analysis

RAW264.7 cells were seeded into a 24‐well plate at a density of 5×10^4^ cells per well, followed by incubation at 37 °C for 24 h. Cells were treated with PBS and 40 µg mL^−1^ DNDA with or without pHLIP modification for another 24 h. For BMDM cells from WT or STING^mut^ mice, cells seeded in a 6‐well plate were treated with DNDA in the same way. Cell medium was collected and centrifuged to remove cell debris for ELISA assays. ELISA assays for IFN‐β and CXCL10 detection were performed according to the manufacturer's protocols. Briefly, all samples and standard samples were added to an IFN‐β or CXCL10 antibody precoated 96‐well plate and incubated at room temperature for 2 h. This was followed by the addition of a biotin‐labeled detection antibody and incubation for another 2 h; streptavidin‐HRP was added and incubated at room temperature for 20 min. The plates were washed three times by washing the solution after each above step. The color‐substrate solution was then added to incubate for another 20 min before the addition of the stop solution. The plates were subjected to a plate reader to detect the absorbance at 450 nm. Standard curves were fitted by four‐parameter Sigmoidal nonlinear regression using GraphPad Prism 8.0 software.

### ELISA—In vivo analysis

Tumors excised from mice were homogenized by RIPA lysis buffer ((Beyotime biotech) supplemented with protease/phosphatase inhibition cocktails (Thermo Fisher Scientific). The samples were then centrifuged at 3000 rpm min^−1^ for 10 min to remove precipitate and stored at −80 °C. For plasma analysis, 200 µL of peripheral blood was collected from C57BL/6 mice in an EDTA‐treated tube. Samples were centrifuged for 30 min at 3000 rpm min^−1^, and the supernatant was collected and stored at −80 °C. Cytokines were analyzed by enzyme‐linked immunosorbent assay (ELISA). The production of cytokines (CXCL‐10, IFN‐β, and TNF‐α) was measured using ELISA kits (Exocell, China) according to the manufacturer's instructions.

### STING Activation and Therapeutic Effect Study of DNDA In Vivo

For STING activation assay in vivo, C57BL/6 and STING^mut^ mice were inoculated with 2 × 10^6^ MC38 tumor cells on the right back. The mice were then randomly grouped into PBS and DNDA‐treated groups (n = 5 in each group) when the tumor reached ≈ 100 mm^3^. The tumors were injected with PBS or 75 µg DNDA every 3 days for comparison evaluation. Mice were injected four times and then sacrificed at a tumor burden endpoint of 20 mm in any dimension. Tumor volume was monitored every 3 days and calculated as length × width × width/2 (length means the longest dimension, width means the shortest dimension). The tumor growth rate was calculated as (1‐V_treated_/V_PBS_) ×100%. Tumors extracted from mice were obtained for further analysis.

### Real‐Time PCR Analysis

In order to perform RT‐PCR analysis of DNDA‐induced STING activation, the relevant mRNA was detected. RAW264.7 were seeded into a 24‐well plate at a density of 5×10^4^ cells per well, followed by incubation at 37 °C for 24 h. After being treated with 0.5 µm DNDA for 24 h, cells were collected. For WT and STING^mut^ mice, after indicated treatment, tumors were extracted 21 days after inoculation. Total RNA was extracted by RNA‐Quick Purification Kit and Tissue RNA purification Kit (Yishan biotech) for cells and tumor tissues respectively, following the manufacturer's procedure. cDNA was synthesized by reverse transcription using Fast Reverse Transcription Kit (Yishan biotech). Then, quantitative Real‐Time PCR was performed using 2× Super SYBR Green qPCR Master Mix (Yishan biotech), and relative quantification was calculated and normalized against GAPDH. The primer sequences for qPCR analysis are listed below.

**Table:** 

mGAPDH	F	GGTTGTCTCCTGCGACTTCA
	R	TGGTCCAGGGTTTCTTACTCC
mIFN‐β	F	CTGGGTGGAATGAGACTATTGT
	R	AAGTTCCTGAAGATCTCTGCTC
mCXCL10	F	CAACTGCATCCATATCGATGAC
	R	GATTCCGGATTCAGACATCTCT
mIsg15	F	AGCGAGCCTCTGAGCATCCTG
	R	GCGTGTCTACAGTCTGCGTCAG

### Flow Cytometry Analysis

Tumors were collected on the 21st day after inoculation. The tissues were made into cell suspension using digesting media containing 1 mg mL^−1^ Collagenase I (Yeasen) and 200 µg mL^−1^ DNase I (Roche) for 30 min at 37 °C. For the staining, single cell suspensions were blocked with TruStain FcX^TM^ (anti‐mouse CD16/32) Antibody (Biolegend, Clone: 93) and stained with antibodies against CD45 (Biolegend, Clone: S18009F), CD3 (Invitrogen, Clone: 145‐2C11), CD8 (Biolegend, Clone: 53–6.7), CD4 (Biolegend, Clone: RM4‐5), CD62L (Biolegend, Clone: MEL‐14), CD44 (Biolegend, Clone: IM7), anti‐mouse CD86 (Invitrogen, Clone: GL1) and dead cells were excluded utilizing Zombie NIR^TM^ Fixable Viability Kit (Biolegend).

For nuclear staining, the cells were fixed and permeabilized with a Foxp3 Fixation/Permeabilization working solution (Biolegend, Cat: 424 401) for 60 min at 4 °C in the dark. The antibody used was anti‐mouse Foxp3 (Biolegend, Clone: FJK‐16s).

For intracellular staining, the cells were fixed with Fixation Buffer (Biolegned, Cat: 420 801) for 20 min at room temperature and permeabilized with Intracellular Staining Permeabilization Wash Buffer (10X) (Biolegend, Cat: 421 002). The antibody used was anti‐mouse CD206 (Invitrogen, Clone: MR6F3).

For cytokine staining, the cells were incubated with Cell Stimulation Cocktail plus protein transport inhibitors (Invitrogen, Cat: 00‐4975‐03) for 4 h at 37 °C before intracellular staining. The antibodies used were anti‐mouse IFNγ (Biolegend, Clone: XMG1.2) and Granzyme B (Biolegend, Clone: GB11). After staining, the cells were then suspended in FACS (PBS and 2% FBS) buffer for flow cytometry analysis. The acquisition was performed on a CytoFLEX flow cytometer, and data were analyzed with CytExpert and FlowJo.

### Cellular Uptake Assay—Co‐culture of RAW264.7 and CT26 cells

RAW264.7 and CT26 cells were seeded into a 24‐well plate at a density of 5×10^4^ cells per well, followed by incubation at 37 °C for 24 h. Cells were treated with 0.3 µm Cy5‐labeled DNDA or DNDA without pHLIP modification for 4 h. For MSR1 inhibition, 5 mg mL^−1^ dextran sulfate was added to RAW264.7 cells for 24 h. Cells were treated with 0.1 µm Cy5‐labeled DNDA and Cy5‐labeled L‐DNDA respectively for 4 h. The medium was then removed, and the cells were washed with PBS three times. RAW264.7 cells were scraped, and CT26 cells were digested by 0.25 Trypsin‐EDTA, followed by suspension into the single‐cell solution by PBS.

The cell suspension was then loaded for flow cytometry (Thermo Fisher Scientific, USA) analysis in the RL1 channel. Cells without any treatment were set as the blank control. The mean fluorescent intensity (MFI) of Cy5 signal was analyzed using FlowJo, v10, software.

### Cellular Uptake Assay—In vivo uptake analysis of Cy5‐labeled DNDA

Mice with an established GFP‐MC38 subcutaneous tumor model were intratumorally injected with 75 µg Cy5‐labeled DNDA. 12 h after injection, the tumor tissues were collected and digested into cell suspensions. The cells were stained with anti‐mouse CD45 (Biolegend, Clone: S18009F), anti‐mouse CD11b (Biolegend, Clone: M1/70) and anti‐mouse F4/80 (Biolegend, Clone: BM8), and dead cells were excluded by Zombie NIR^TM^ Fixable Viability Kit (Biolegend). Cy5^+^ cells in tumors were assessed by flow cytometry, and data were analyzed with CytExpert and FlowJo.

### Immunofluorescence

Tumors were embedded in Tissue‐Tek OCT compound (Sakura Finetek) at −80 °C for cryosectioning. The slices were blocked in 10% of FBS for 30 min at room temperature, followed by incubation with PE anti‐mouse F4/80 (Invitrogen, Clone: BM8) in the dark at 4 °C overnight. After removing the first antibody, slices were countered with DAPI (Sigma) for 15 min in the dark at room temperature. Slices were observed by Leica SP8 laser scanning microscope.

### Combinational Therapy of DNDA and Anti‐PDL1 in MC38 Mouse Model

To establish a subcutaneous tumor model, C57BL/6 mice were inoculated with 2×10^6^ MC38 tumor cells on the right back. The mice were randomly grouped (n = 5 per group) and treated with DNDA locally on day 6, followed by i.p. injection of anti‐PDL1 the next day, and repeated on day 9 and day 12: (1) PBS; (2) DNDA (75 µg G3×3, intratumoral injection); (3) αPDL1 (100 µg×3, intraperitoneal injections); (4) DNDA (75 µg G3×3, intratumoral injection) + αPDL1(100 µg×3, intraperitoneal injections). Tumors were excised on day 15 for flow cytometry, Elisa, histologic analysis, and H&E staining.

### Combinational Therapy of DNDA and Anti‐PDL1 in B16 Mouse Model

To establish a subcutaneous tumor model, C57BL/6 mice were inoculated with 2×10^6^ B16 tumor cells on the right back. The mice were randomly grouped (n = 5 per group) and treated with DNDA locally on day 6, followed by i.p. injection of anti‐PDL1 the next day, and repeated on day 9 and day 12: (1) PBS; (2) Free G3 (75 µg×3, intratumoral injection); (3) DNDA (no pHLIP) (75 µg G3×3, intratumoral injection); (4) αPDL1 (100 µg×3, intraperitoneal injections); (5) DNDA (75 µg G3×3, intratumoral injection) and (6) DNDA (75 µg G3×3, intratumoral injection)+αPDL1 (100 µg×3, intraperitoneal injections). When 1D of the tumor reached 20 mm, the mice were sacrificed for tumor acquisition.

### Statistical Analysis

Data are presented as Mean ± standard deviation (SD) for all results from at least three repeated experiments. Statistical significance was assessed using one‐way analysis of variance (ANOVA) or two‐way ANOVA for multiple comparisons, and a two‐tailed unpaired *t*‐test was used for comparing two groups. Survival was analyzed using Kaplan‐Meier survival curves, and the curves were compared with the log‐rank Mantel‐Cox test. *p* < 0.05 was considered significant, the significance levels are ^*^ *p* < 0.05, ^**^ *p* < 0.01, ^***^ *p* < 0.001, and ns means no significance. GraphPad Prism 8.0 was used for statistical analysis.

## Conflict of Interest

The authors declare no conflict of interest.

## Supporting information

Supporting Information

## Data Availability

The data that support the findings of this study are available from the corresponding author upon reasonable request.
