# Supplementary material for: A DNA‐Modularized STING Agonist with Macrophage‐Selectivity and Programmability for Enhanced Anti‐Tumor Immunotherapy
Source: Adv Sci (Weinh). 2024 Jun 19;11(32):2400149. doi: 10.1002/advs.202400149 (PMC11348061; doi:10.1002/advs.202400149)
Supplement: Supplementary file 1 — Supporting Information [file ADVS-11-2400149-s001.docx]

Supporting Information

A DNA-modularized STING agonist with macrophage-selectivity and programmability for enhanced anti-tumor immunotherapy

*Yingzhi Chen, Ruike Li, Qiao Duan, Lingling Wu, Xinyi Li, Aoxiang Luo, Yongming Zhang, Na Zhao, Kai Cui, Wenwei Wu, Tize Liu, Jian-Bo Wan, Liufu Deng, Guiying Li*, Lijun Hou*, Weihong Tan*, and Zeyu Xiao**

Y. Chen, R. Li and Q. Duan contributed equally to this work.

Y. Chen, R. Li, X. Li, A. Luo, Y. Zhang, N. Zhao, K. Cui, W. Wu, T. Liu, Z. Xiao

Department of Pharmacology and Chemical Biology, Key Laboratory of Cell Differentiation and Apoptosis of Chinese Ministry of Education, Shanghai Jiao Tong University School of Medicine, Shanghai 200025, China

Y. Chen, Q. Duan, W. Tan, Z. Xiao

Institute of Molecular Medicine, Shanghai Key Laboratory of Nucleic Acid Chemistry and Nanomedicine, Renji Hospital, Shanghai Jiao Tong University School of Medicine, Shanghai, 200127, China

L. Wu, L. D

Shanghai Institute of Immunology, Department of Immunology and Microbiology, Shanghai Jiao Tong University School of Medicine, Shanghai 200025, China

1. Hou

Department of Neurosurgery, Changzheng Hospital, Naval Medical University, Shanghai, 200003, China

1. Li

Department of Nephrology, the Affiliated Hospital of Hebei Engineering University, Hebei, 056029, China

J. Wan

State Key Laboratory of Quality Research in Chinese Medicine, Institute of Chinese Medical Sciences, University of Macau, Taipa, Macau SAR, China.

E-mail: [fflgy@126.com](mailto:liguiying645@163.com) (G. Li), [lijunhoucz@126.com](mailto:lijunhoucz@126.com) (L. Hou), [tan@hnu.edu.cn (W. Tan),](mailto:wtan@hnu.edu.cn,) [zxiao@sjtu.edu.cn](mailto:zxiao@sjtu.edu.cn) (Z. Xiao)


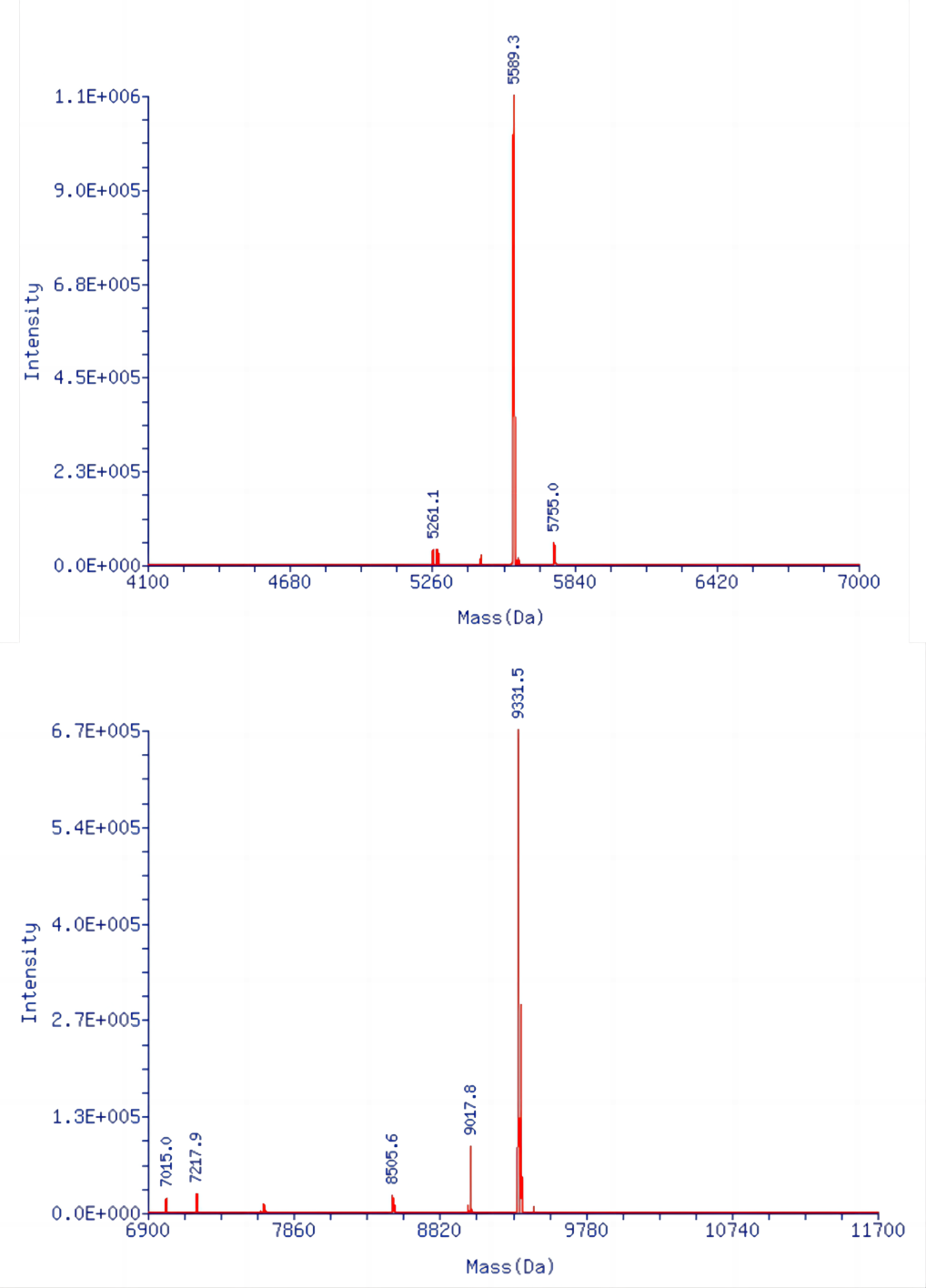


**Figure S1.** LC/MS spectrum of Yl and Yl-p conjugate.


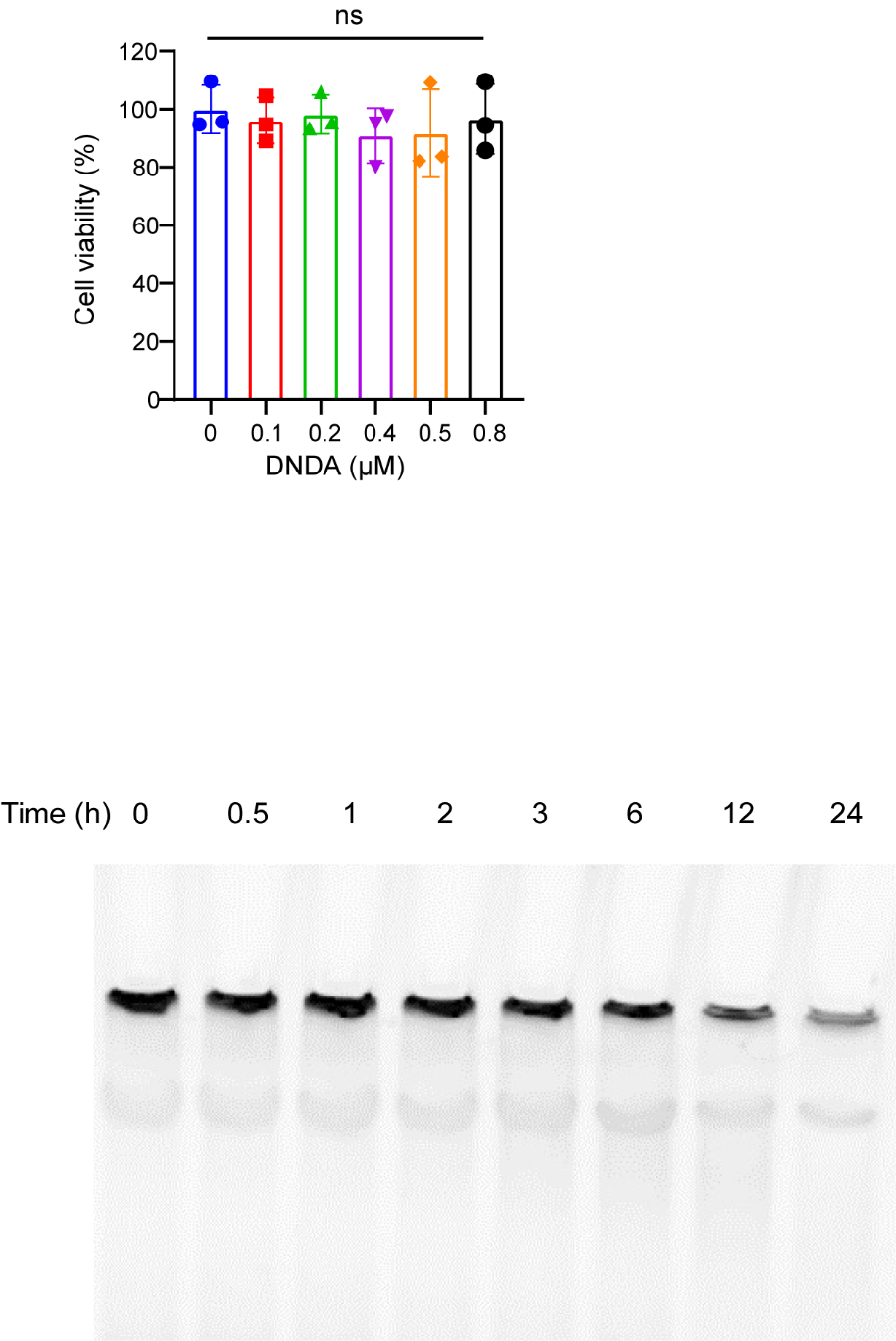


**Figure S2.** The cytotoxicity of DNDA in RAW264.7 cells incubated with indicated concentration of DNDA. Data are shown as the Mean ± SD (n = 3), statistical significance was calculated via one-way ANOVA with Tukey’s post hoc test, ns means no significance.


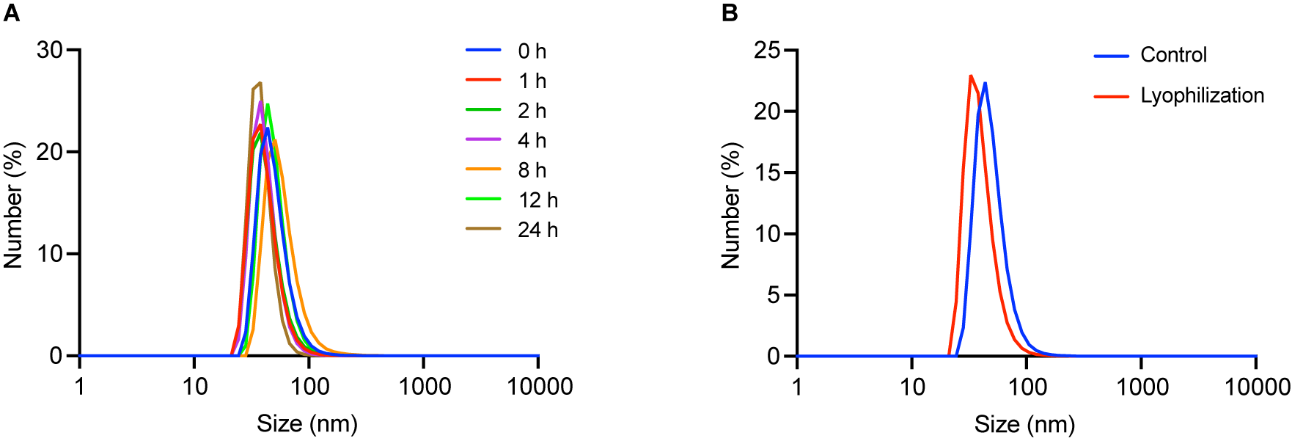


**Figure S3.** Serum stability and storability of DNDA. (A) The particle size of DNDA after incubation in 10% FBS for the indicated times. (B) The particle size of DNDA before and after lyophilization.


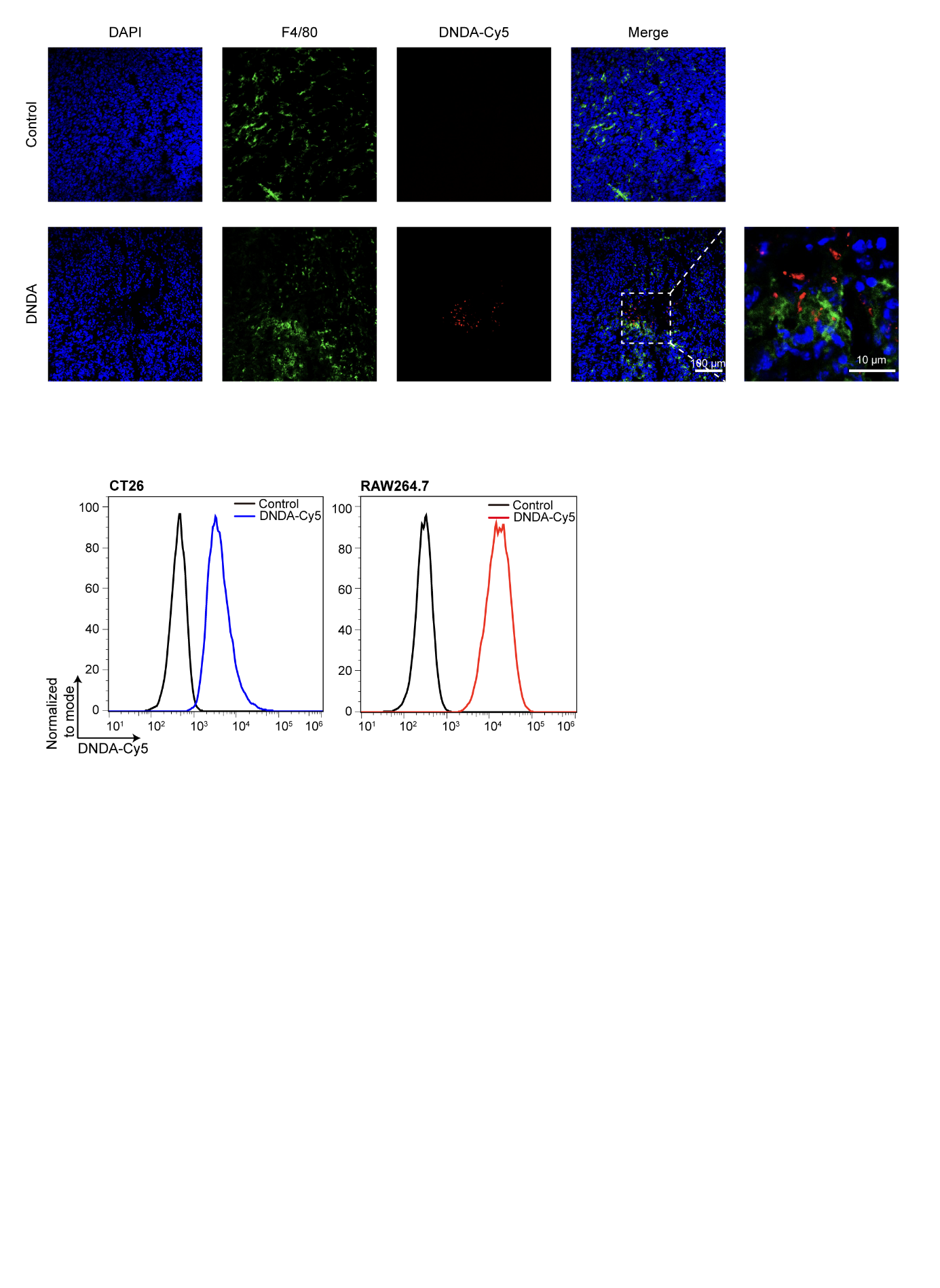


**Figure S4.** Cellular uptake level of CT26 and RAW264.7 when treated with Cy5-labeled DNDA.


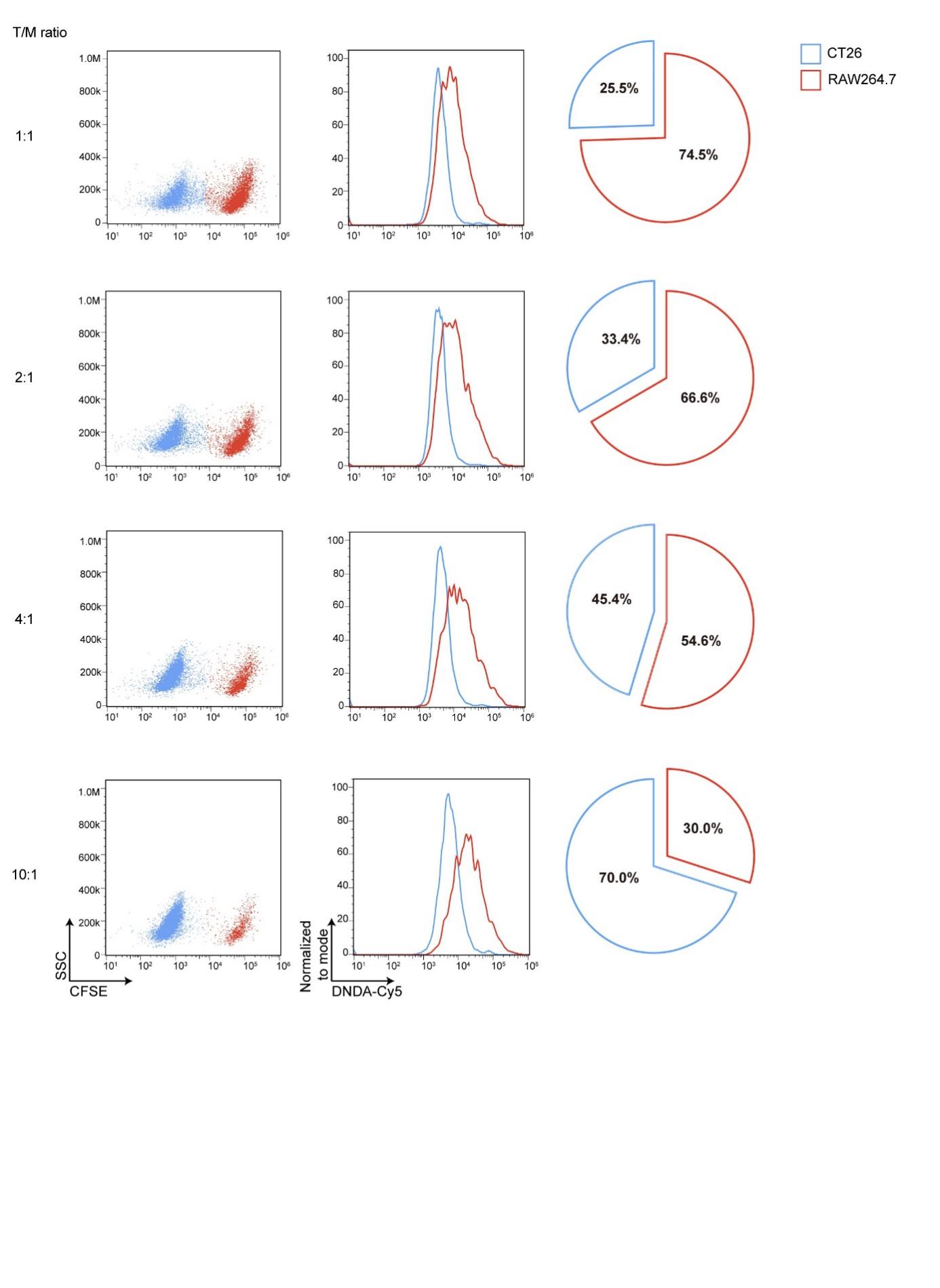


**Figure S5.** Macrophage-selective uptake of DNDA. The quantitative MFI analysis of Cy5-labeled DNDA uptake level (left panel) and ratio (right panel) in CT26 and RAW264.7 cells by flow cytometry in CT26/RAW264.7 coculture model with indicated T/M ratio.


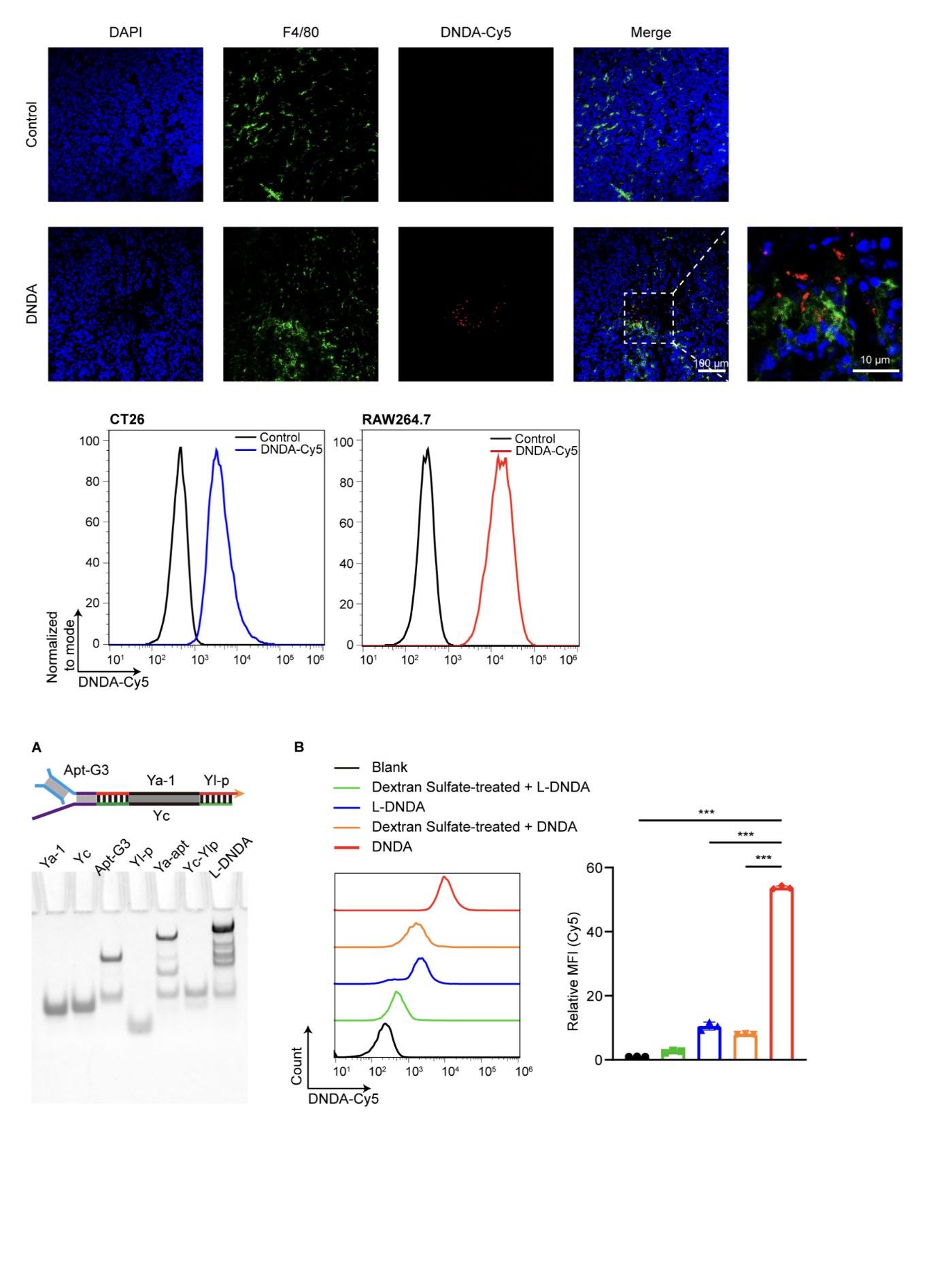


**Figure S6.** The RAW264.7 cell uptake of DNDA and linear DNDA (L-DNDA) with or without MSR1 blockade. (A) Design and synthesis of L-DNDA. (B) Quantitative Cy5 MFI analysis in RAW264.7 cells with different treatments. Data are shown as the Mean ± SD (n = 3), statistical significance was calculated via one-way ANOVA with Tukey’s post hoc test, *** *p* < 0.001.


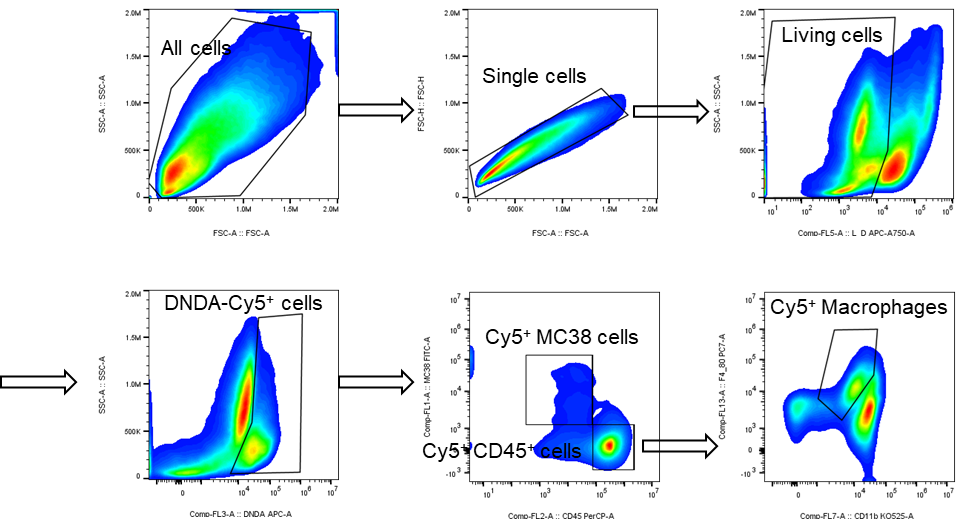


**Figure S7.** Gating strategy of Cy5^+^ cells, Cy5^+^ MC38 tumor cells and Cy5^+^ macrophages in MC38-GFP tumors.


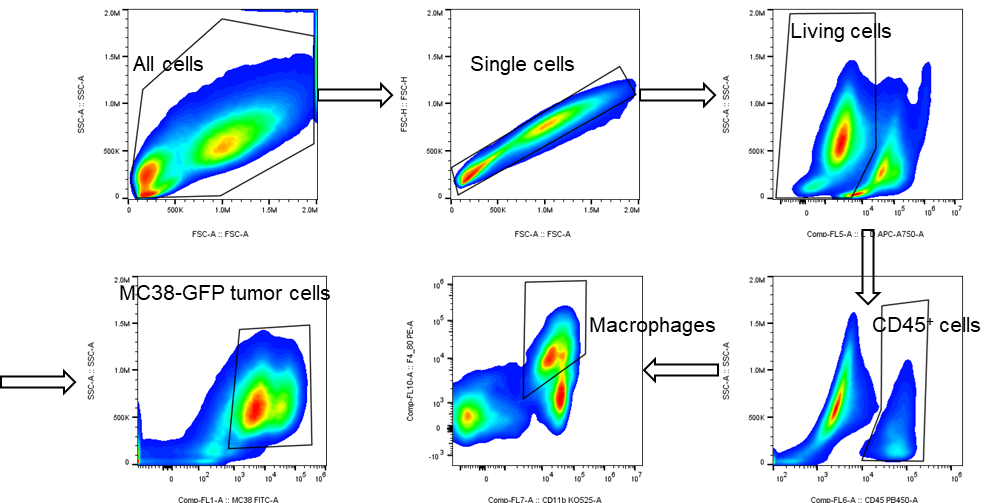


**Figure S8.** Gating strategy of MC38-GFP tumor cells and macrophages in MC38-GFP tumors.


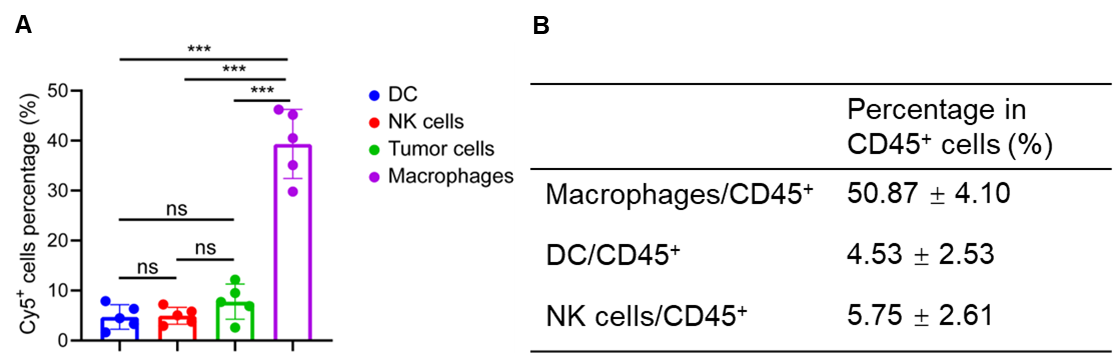


**Figure S9.** Holistic assessment of tumor microenvironment after i.t. injection of Cy5-labeled DNDA. (A) Percentage of DNDA-Cy5^+^ DCs, DNDA-Cy5^+^ NK cells and DNDA-Cy5^+^ macrophages after injected with Cy5-labeled DNDA in MC38-GFP tumor-bearing mice. Data are shown as Mean ± SD (n = 5), statistical significance was calculated via one-way ANOVA with Tukey’s post hoc test, ****p* < 0.001, ns means no significance. (B) The proportion of macrophages, DCs and NK cells among CD45^+^ cells. Data are shown as the Mean ± SD (n = 5).


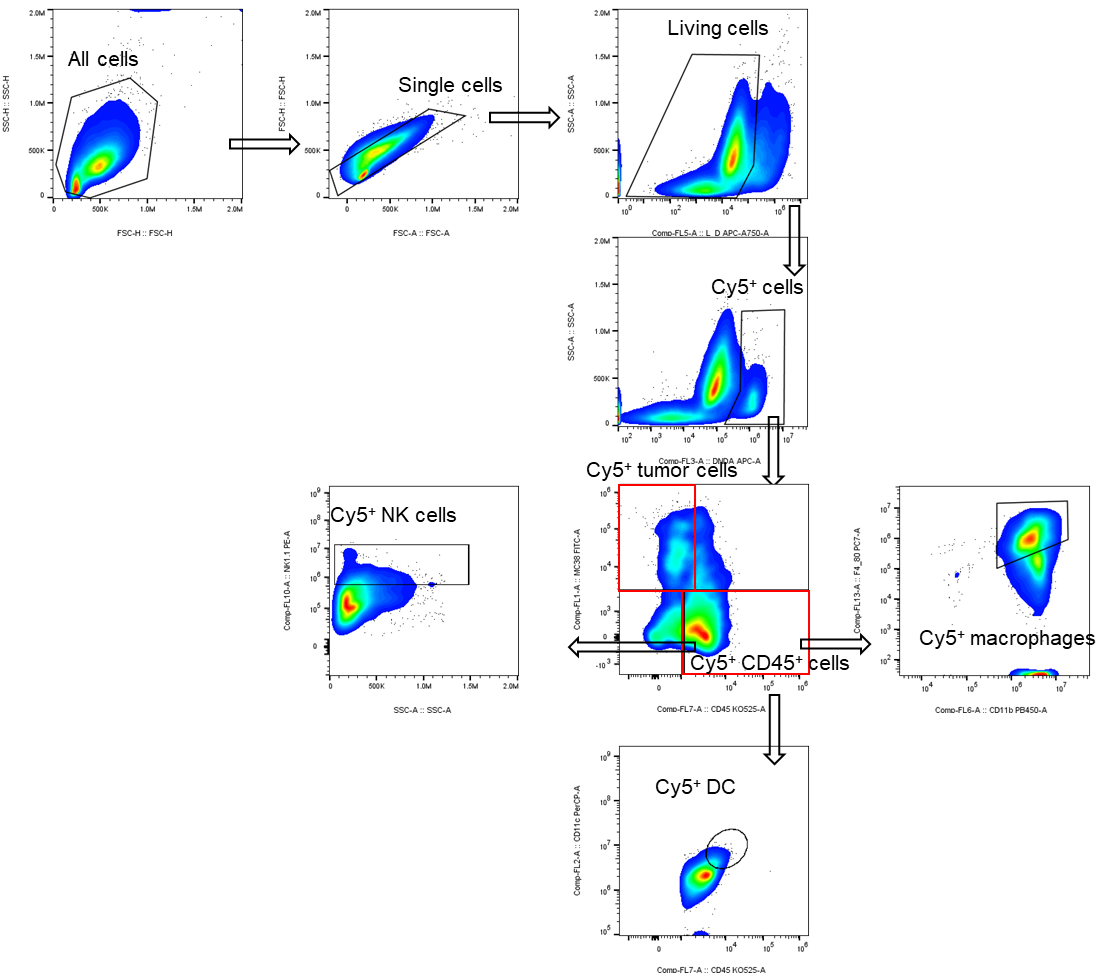


**Figure S10.** Gating strategy of Cy5^+^ cells, Cy5^+^ tumor cells, Cy5^+^ macrophages, Cy5^+^ DC and Cy5^+^ NK cells.


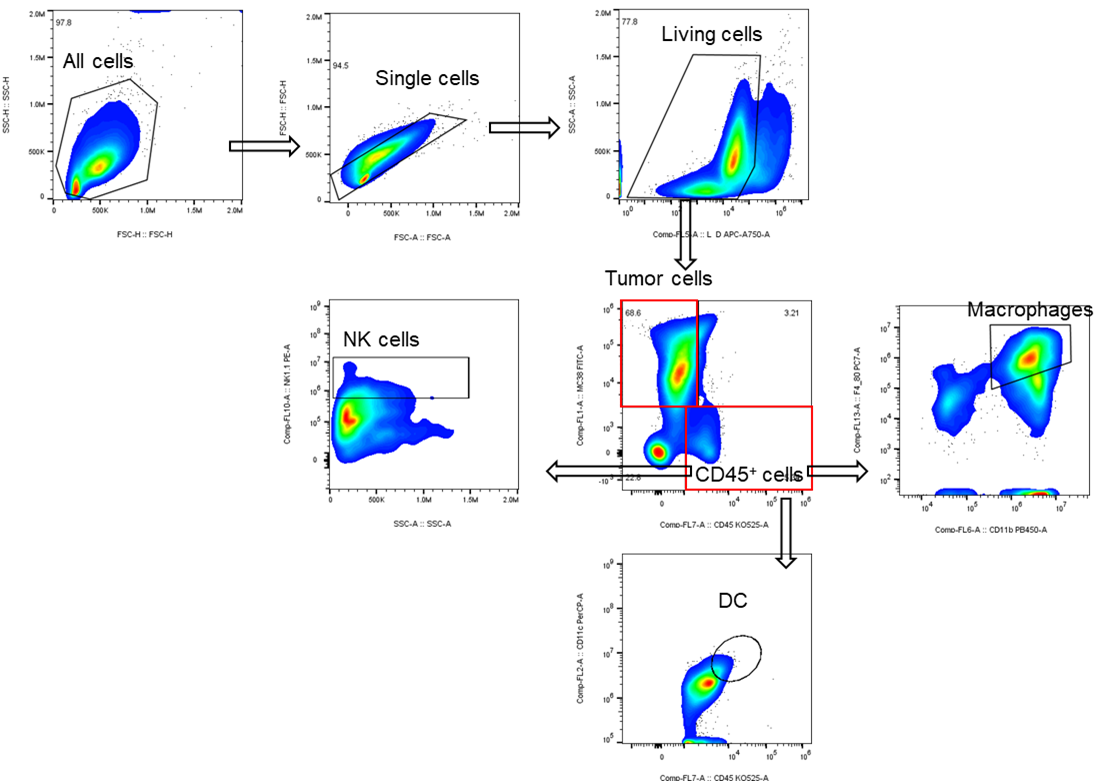


**Figure S11.** Gating strategy of macrophages, DCs and NK cells in tumor microenvironment.


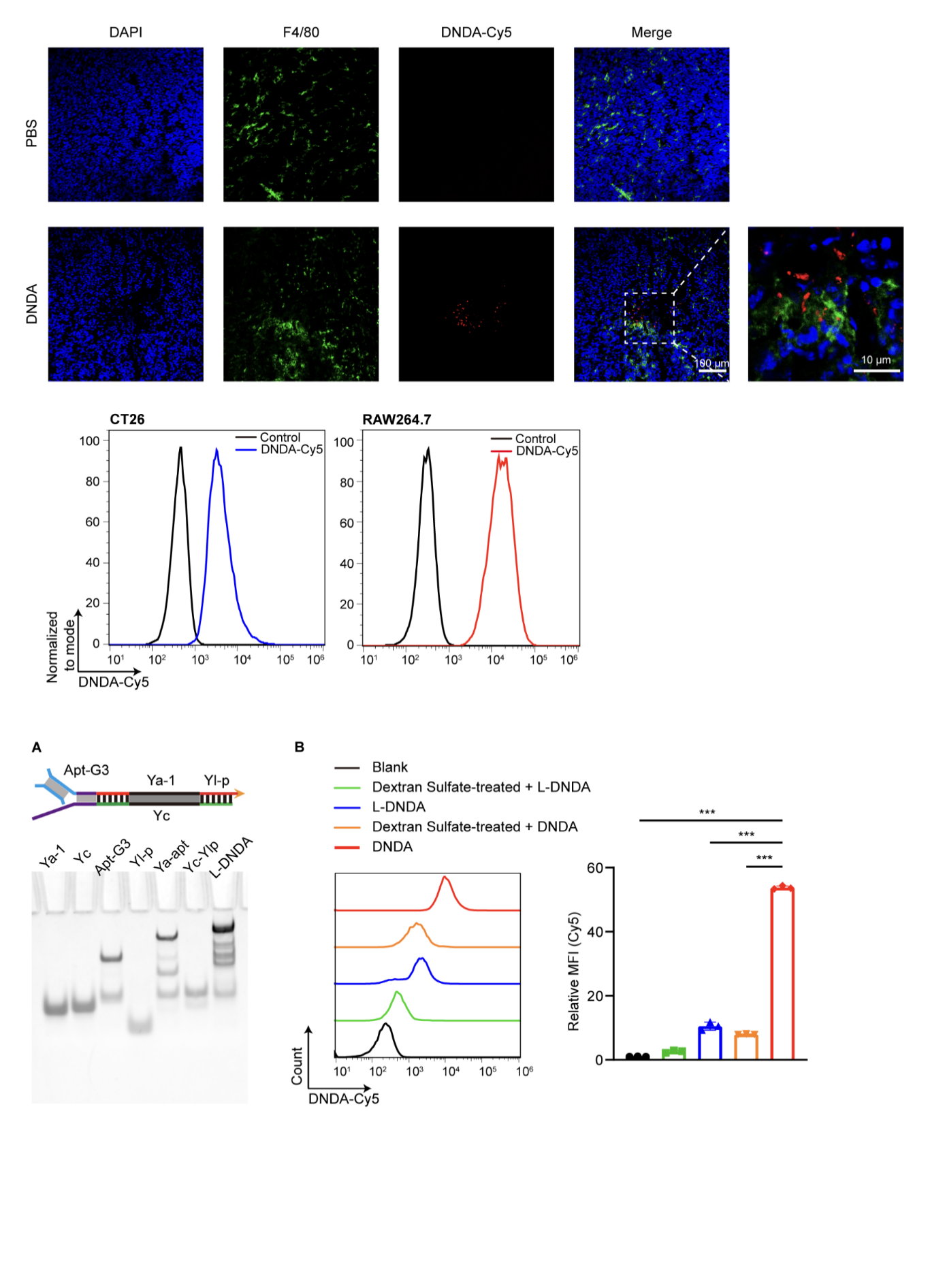


**Figure S12.** Macrophage uptake Cy5-labeled DNDA *in vivo* imaged by LSCM.


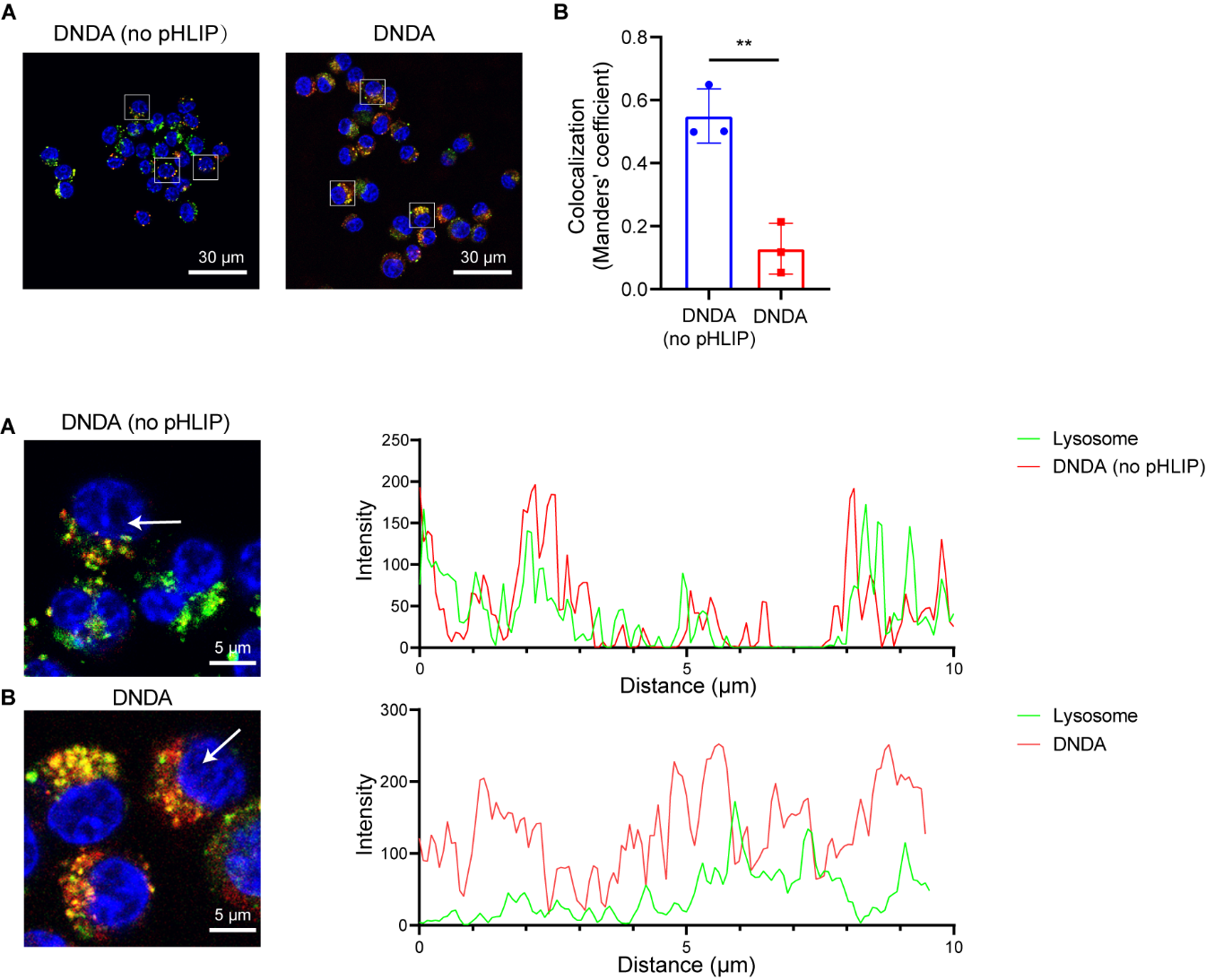


**Figure S13.** Co-localization of DNDA or DNDA (no pHLIP) with lysosomes by (A) LSCM and (B) Mander’s coefficient analysis (representative of three individual cells). Data are shown as Mean ± SD (n = 3), statistical significance was calculated via two-tailed unpaired *t*-test, ***p* < 0.01.


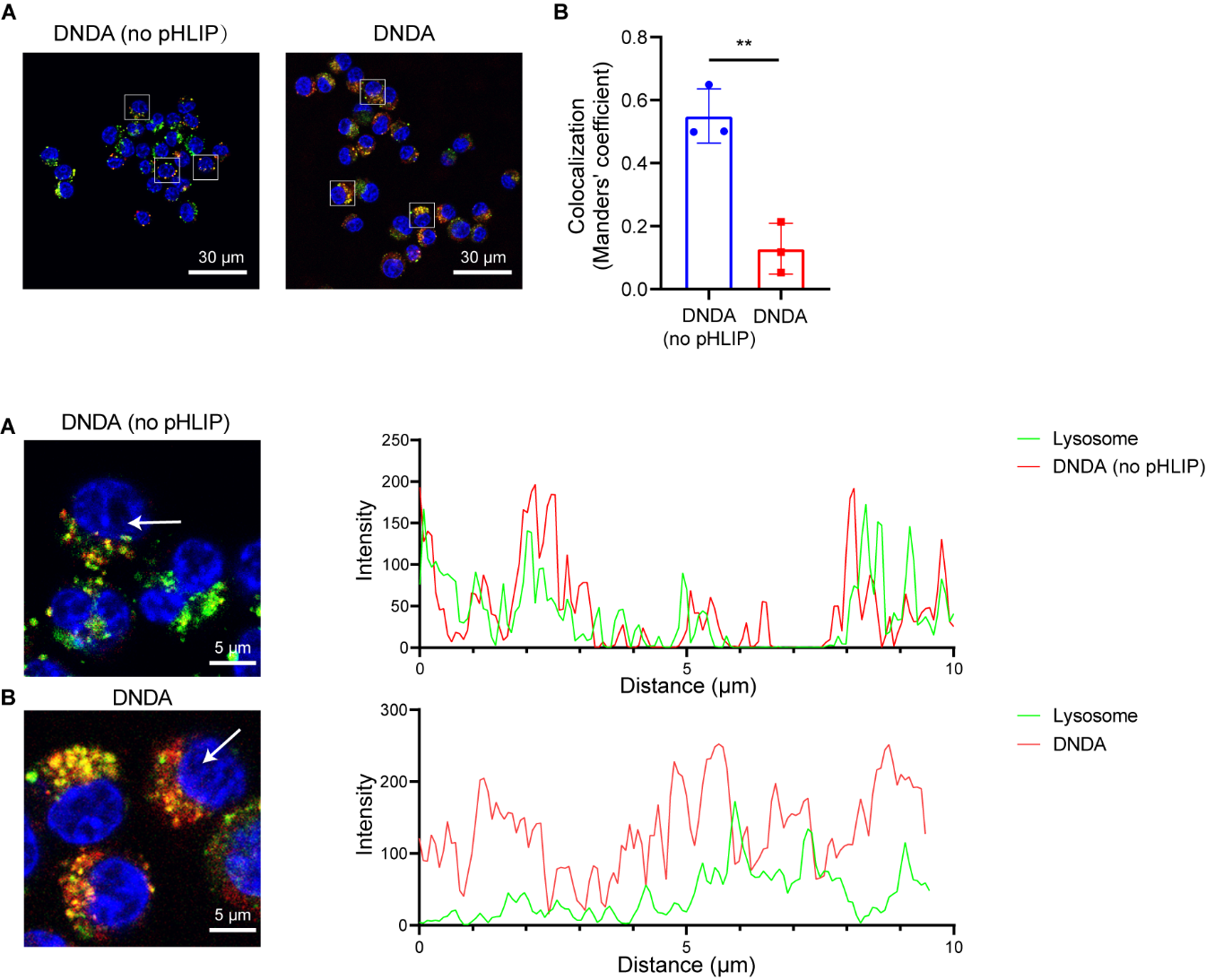


**Figure S14.** The line scan profiles of the fluorescence intensities at the white arrows in DNDA and DNDA (no pHLIP) group, respectively.


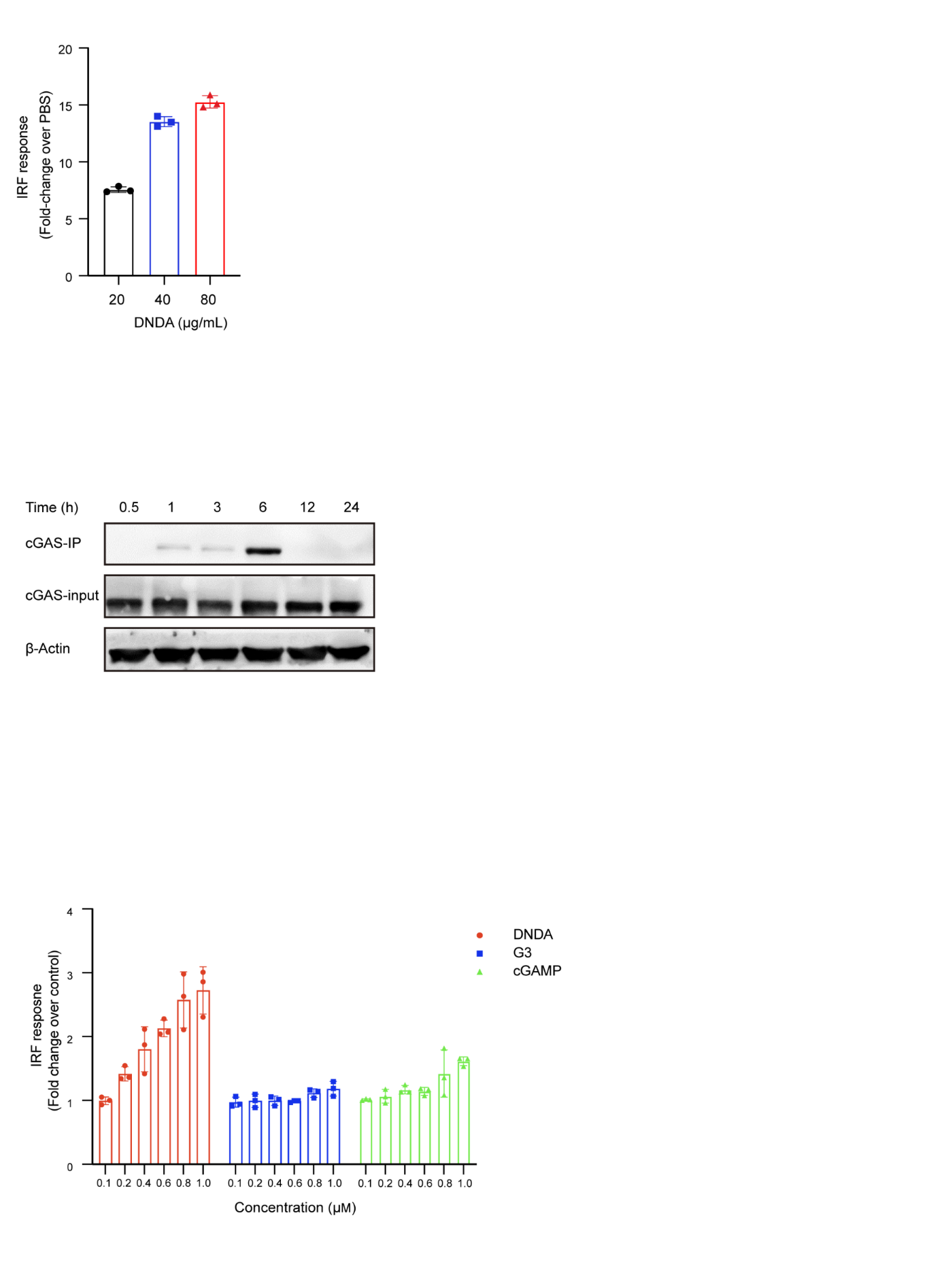


**Figure S15.** The binding and dissociation process between G3-biotin and cGAS after co-incubated for indicated time using co-IP analysis.


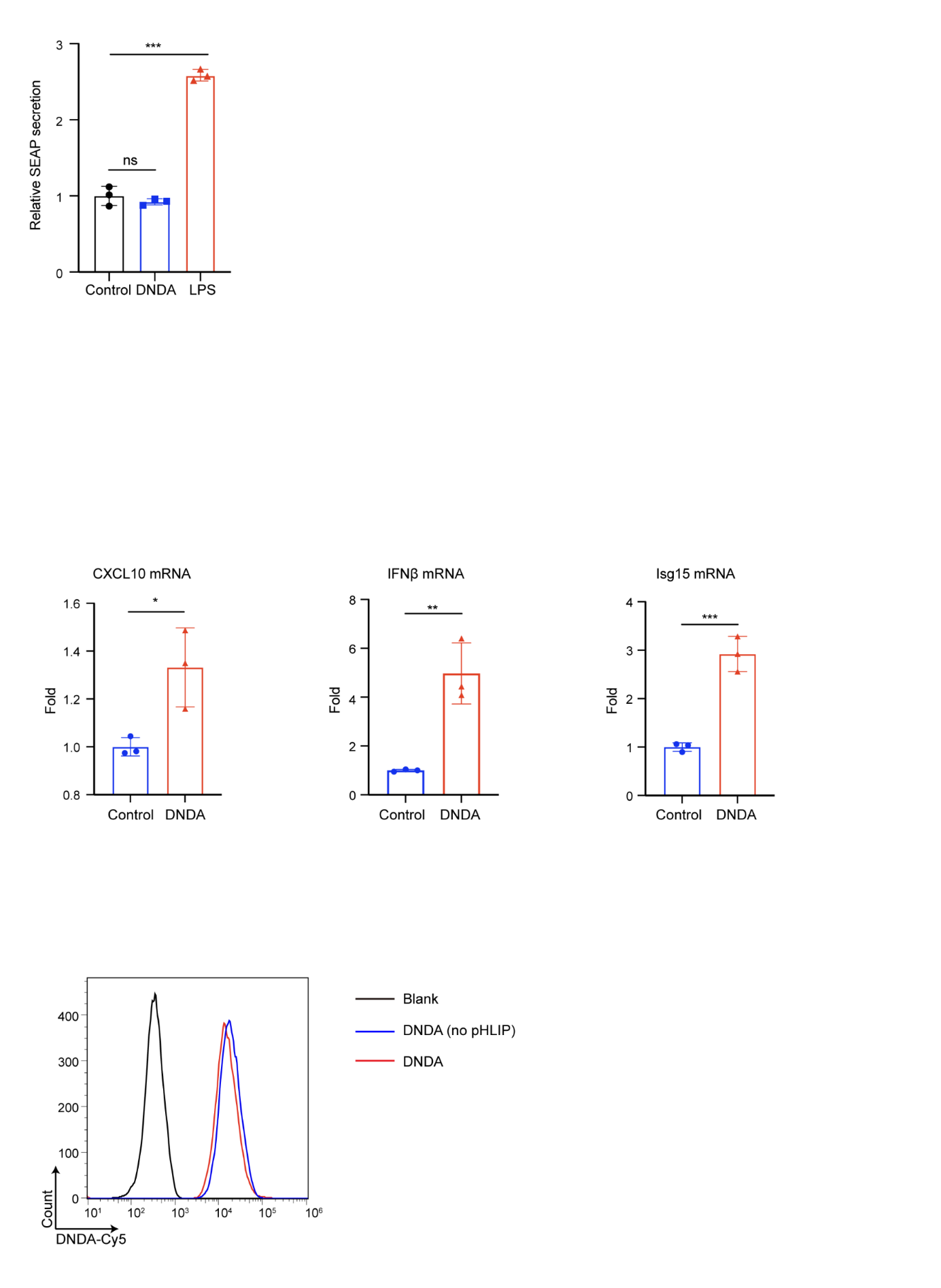


**Figure S16.** Quantitative Cy5 MFI analysis in RAW ISG cells after treated with Cy5-labeled DNDA with or without pHLIP modification.


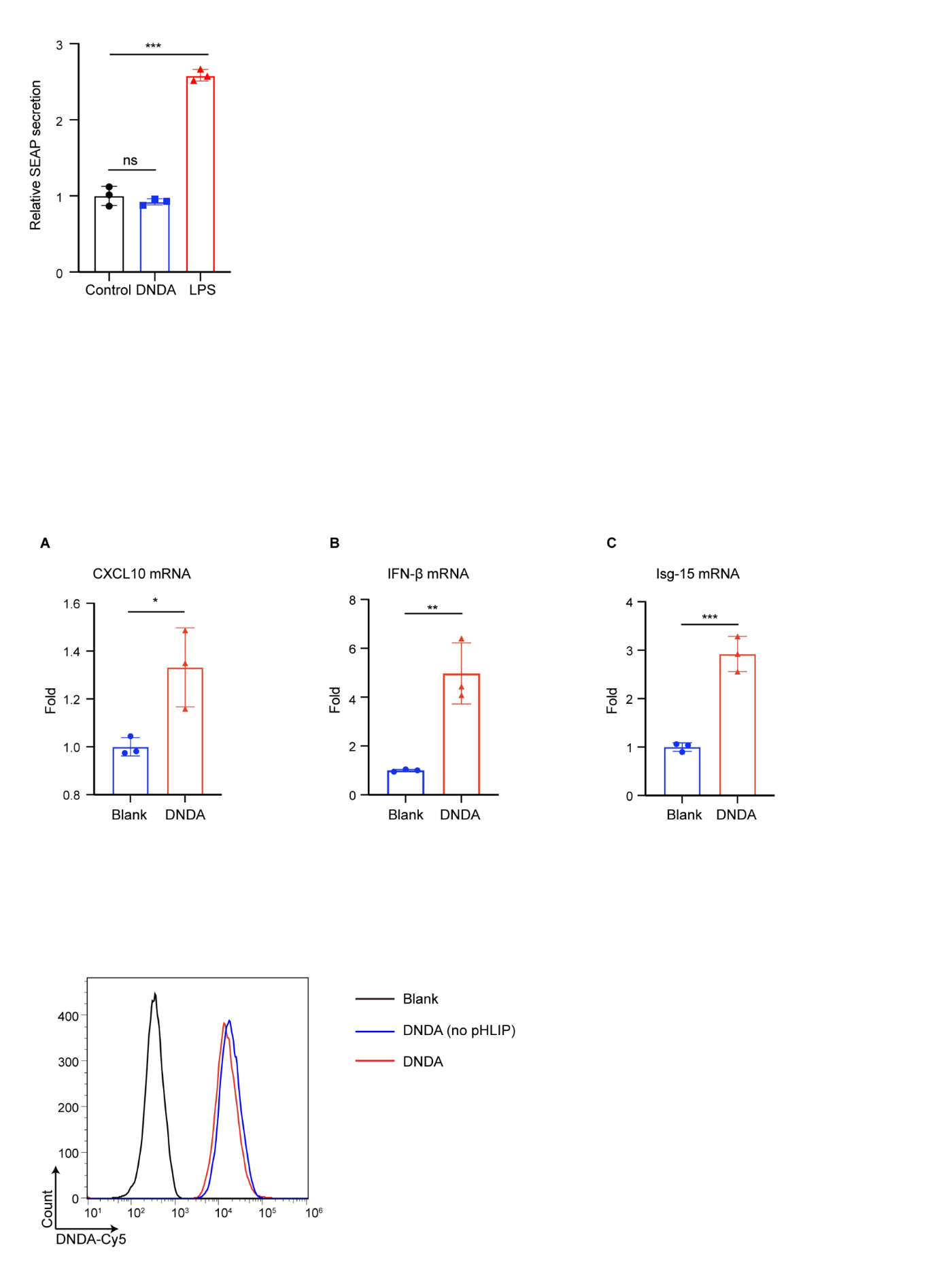


**Figure S17.** Real-Time PCR examination of mRNA transcription of (A) CXCL10, (B) IFN-β and (C) Isg-15 in RAW264.7 cells 12 h post treatment. Data are shown as Mean ± SD (n = 3), statistical significance was calculated via two-tailed unpaired *t*-test, **p* <0.05, ***p* < 0.01, ****p* < 0.001.


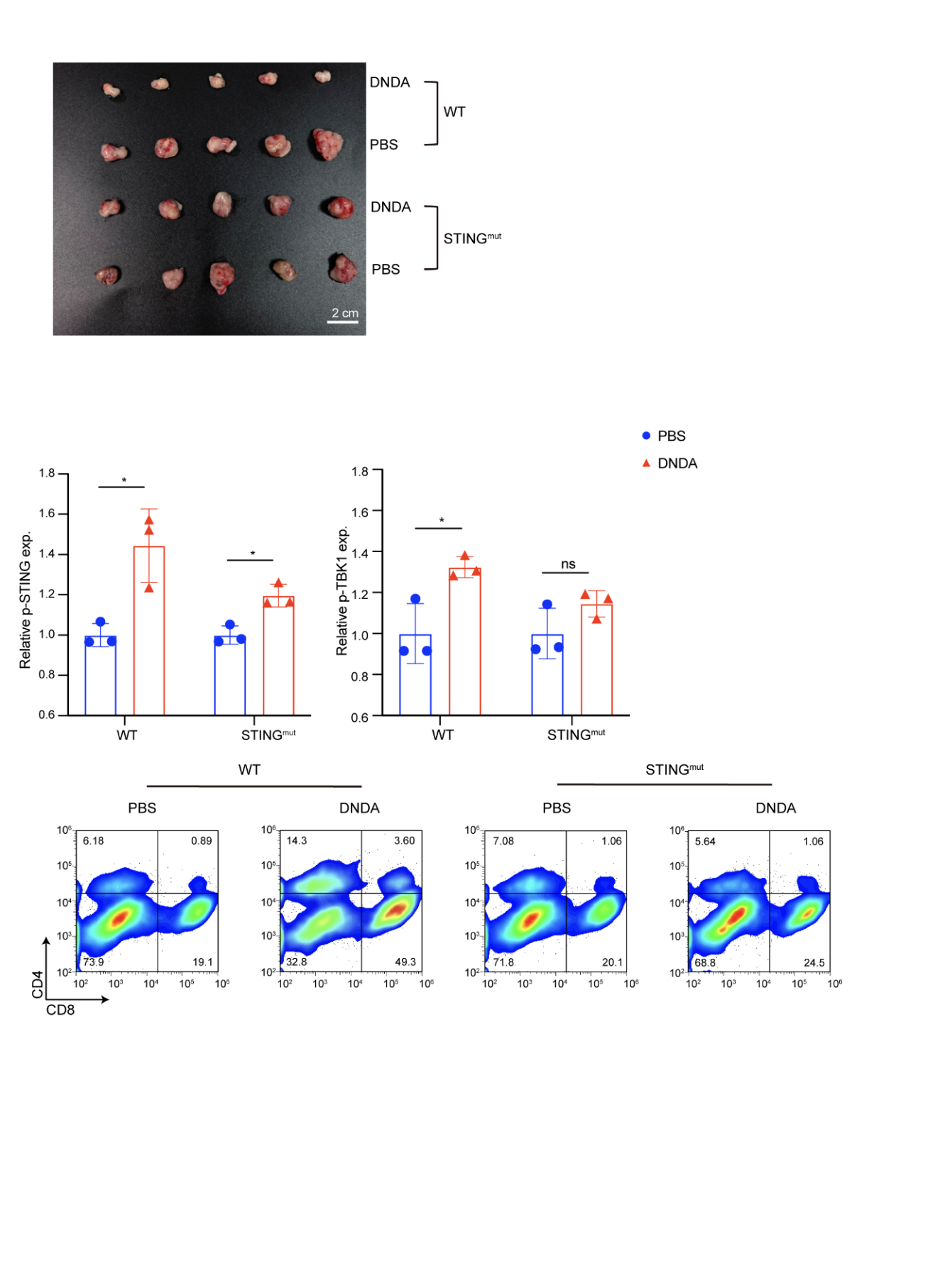


**Figure S18.** Photograph of the tumors extracted from mice 21 days after tumor inoculation. (n = 5)


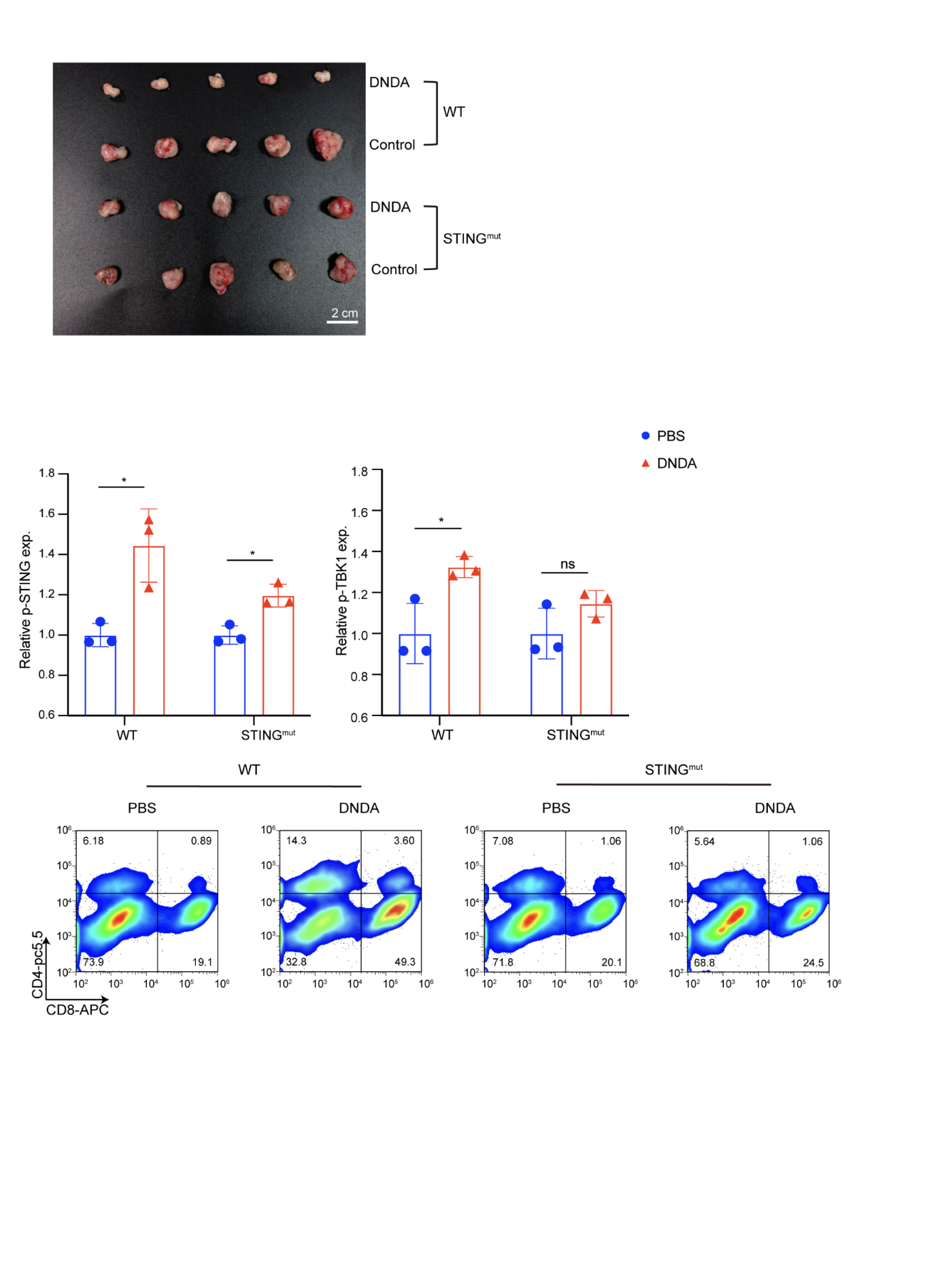


**Figure S19.** The quantification of western blot of STING and TBK1 phosphorylation in tumor tissues. Data are shown as the Mean ± SD (n = 3), statistical significance was calculated via two-way ANOVA with Sidak’s post hoc test, **p* < 0.05, ns means no significance.


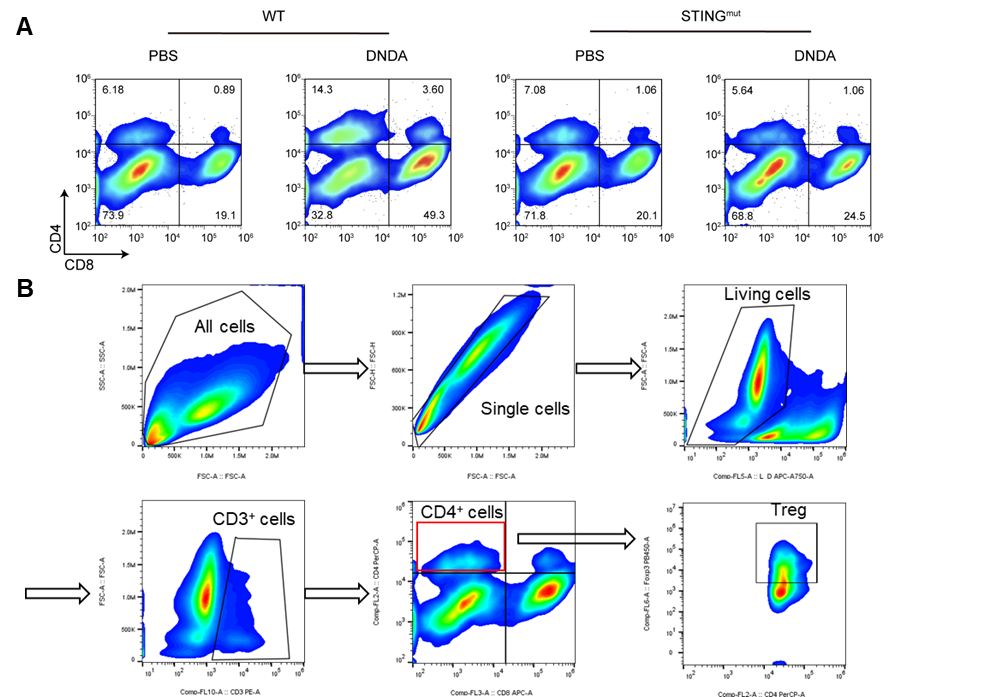


**Figure S20.** (A) Representative flow cytometric plots of CD8^+^ T cells (gated on L/D^-^ CD3^+^ CD8^+^) and CD4^+^ T cells (gated on L/D^-^ CD3^+^ CD4^+^). (B) Gating strategy of CD4^+^ T cells, CD8^+^ T cells and Treg.


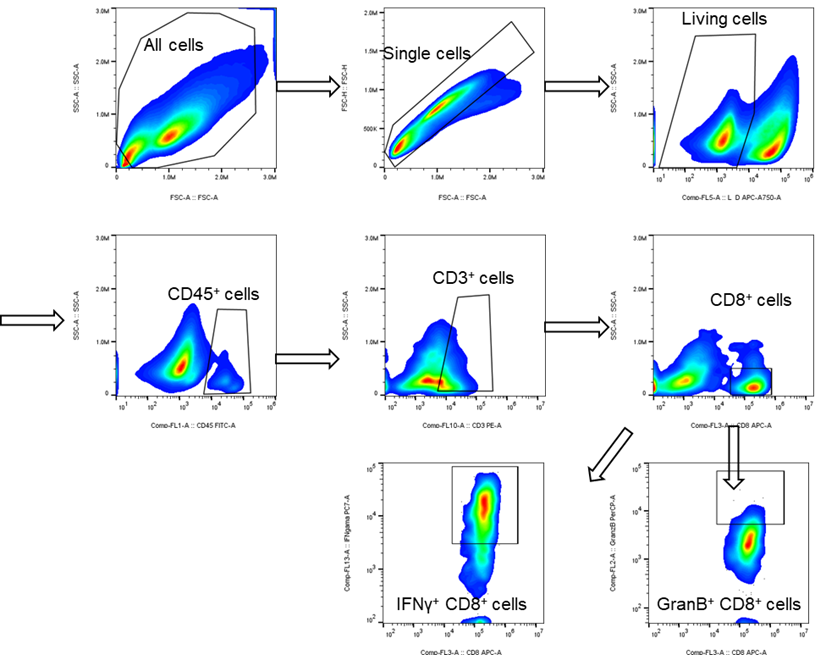


**Figure S21.** Gating strategy of IFNγ^+^ CD8^+^ T cells and GranB^+^ CD8^+^ T cells.


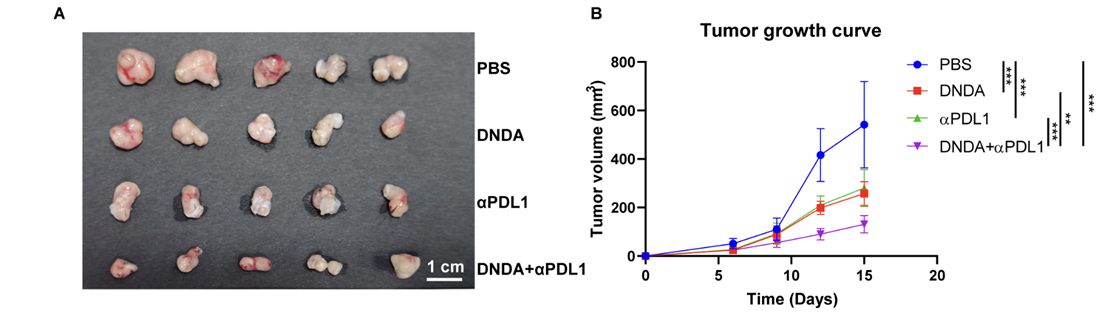


**Figure S22.** Therapeutic effects of DNDA on inhibiting tumor growth combined with PD-1/PD-L1 blockade**.** (A) Photograph of the tumors extracted from mice 15 days after tumor inoculation. (B) Tumor growth curve of MC38 tumor-bearing mice with indicated treatment. Data are shown as the Means ± SD (n = 5), statistical significance was calculated via two-way ANOVA with Tukey’s post hoc test, ****p* < 0.001, ***p* < 0.01.


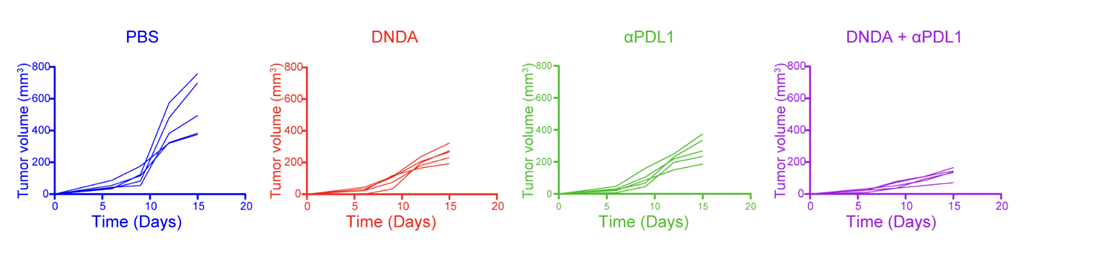


**Figure S23.** Individual tumor growth curve of MC38 tumor-bearing mice. (n = 5)


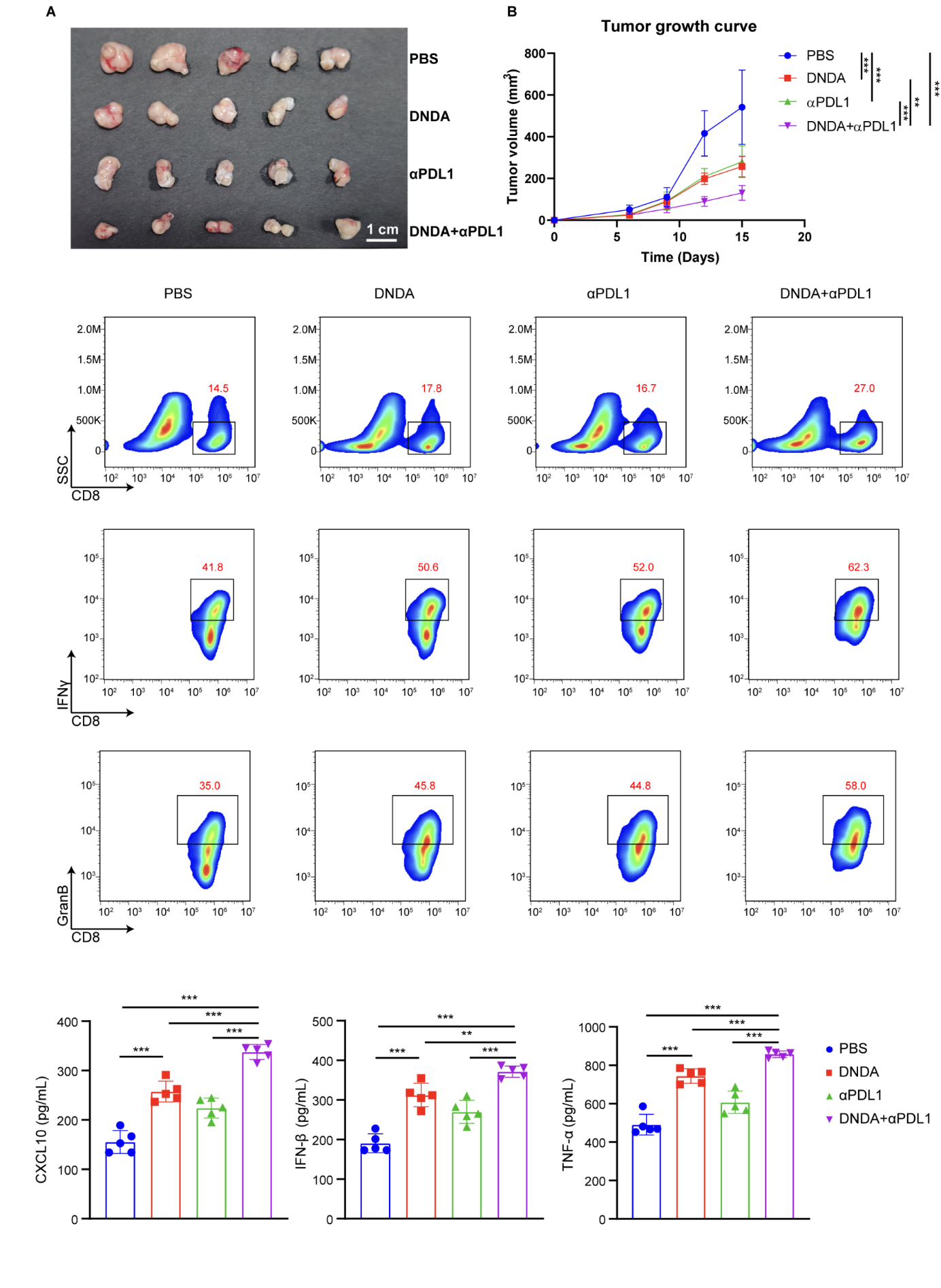


**Figure S24.** Representative flow cytometric plots of CD8^+^ T cells (gated on L/D^-^CD45^+^ CD3^+^ CD8^+^), IFNγ^+^ CD8^+^(gated on L/D CD45^+^ CD3^+^ CD8^+^ IFNγ^+^) and GranB^+^ CD8^+^ T cells (gated on L/D^-^ CD45^+^ CD3^+^ CD8^+^ Granzyme B^+^).


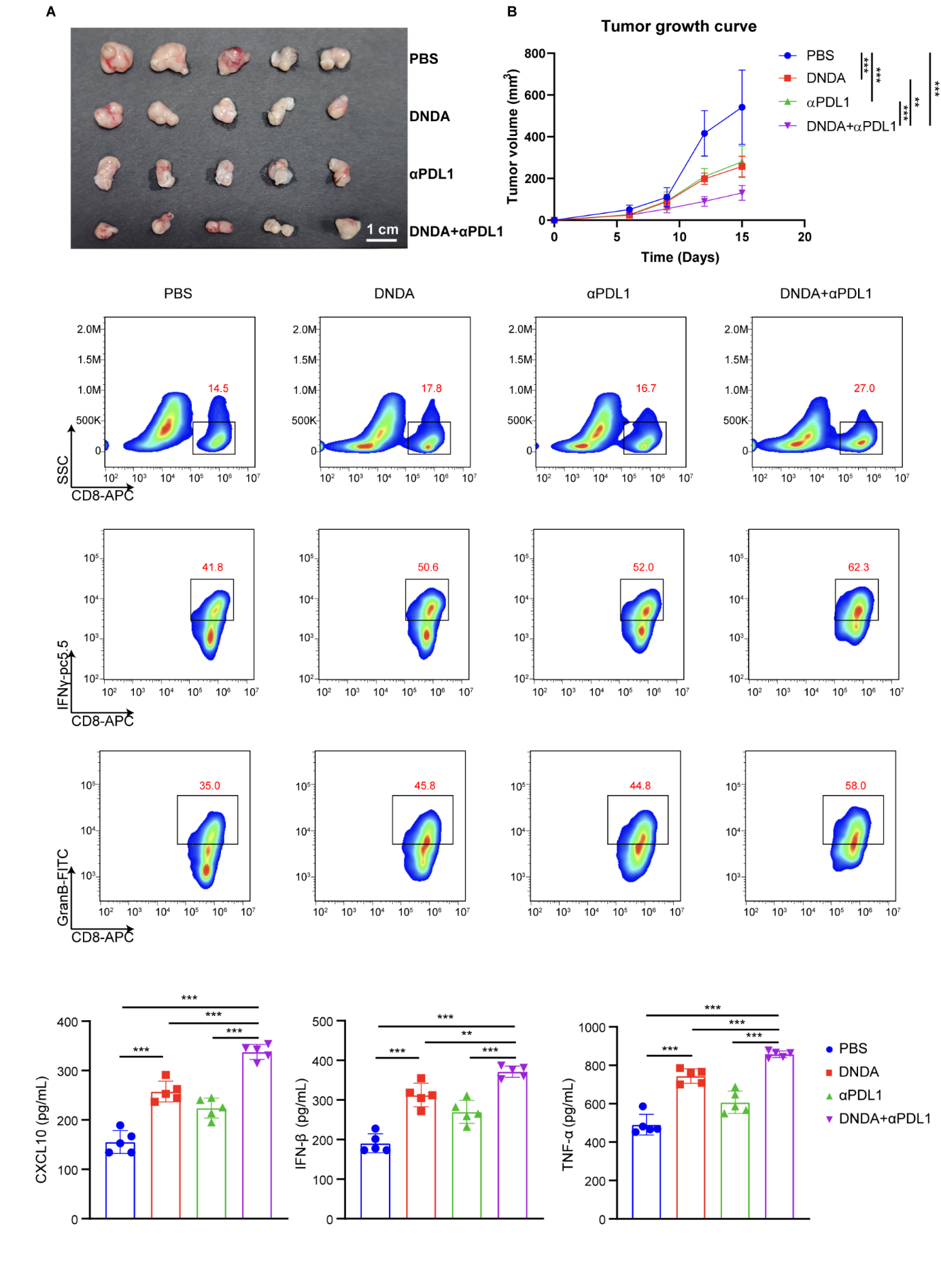


**Figure S25.** ELISA analysis of CXCL10, IFN-β and TNF-α concentration in plasma. Data are shown as Mean ± SD (n = 5), statistical significance was calculated via one-way ANOVA with Tukey’s post hoc test, ***p* < 0.01, ****p* < 0.001.


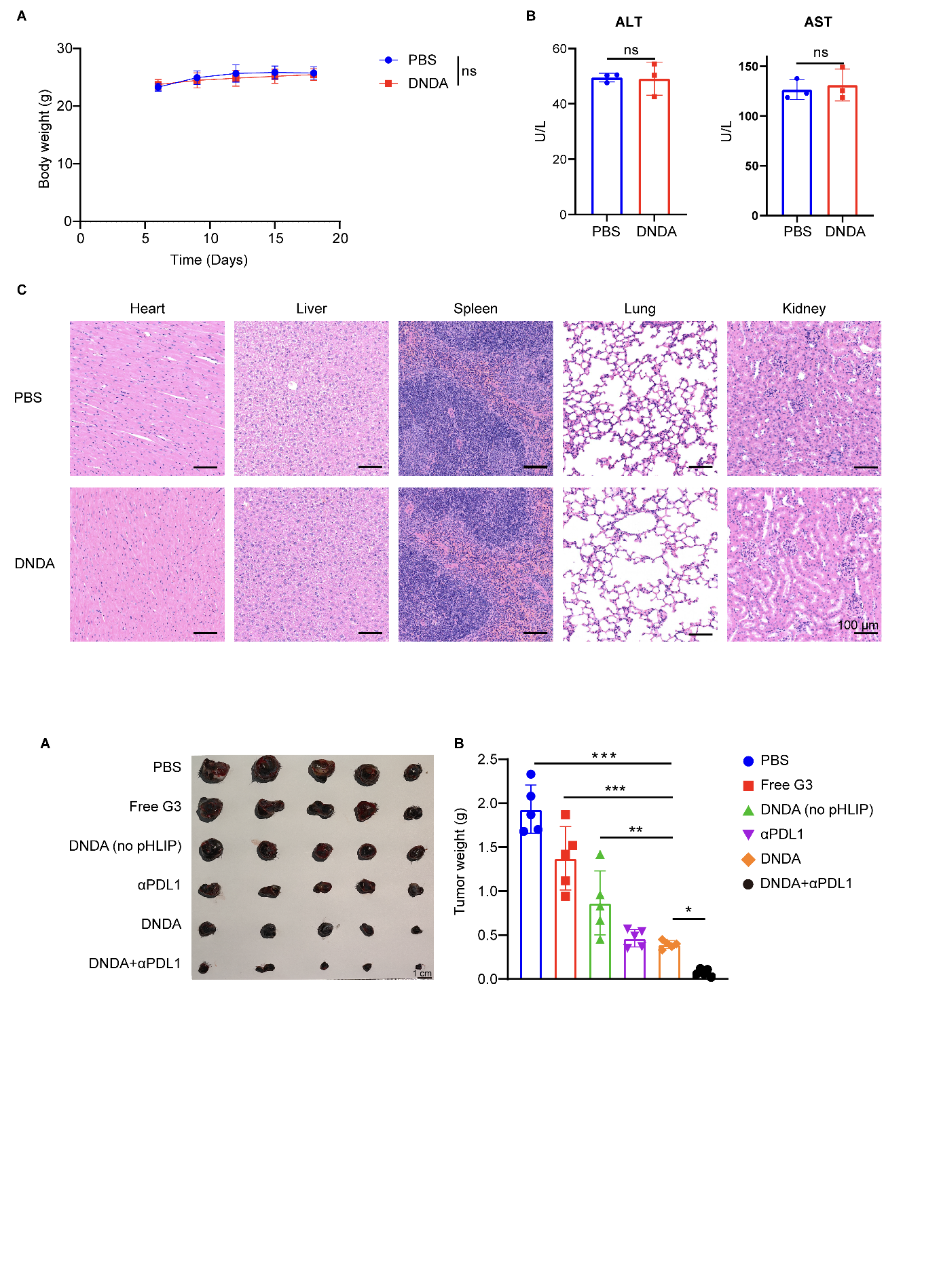


**Figure S26.** Therapeutic effect of combination therapy of DNDA and αPDL1 in B16 tumor-bearing C57BL/6 mice. (A) Photograph of the tumors and (B) tumor weight extracted from mice 18 days after tumor inoculation. Data are shown as Mean ± SD (n = 5), statistical significance was calculated via one-way ANOVA with Tukey’s post hoc test, **p* < 0. 05, ***p* < 0.01, ****p* < 0.001.


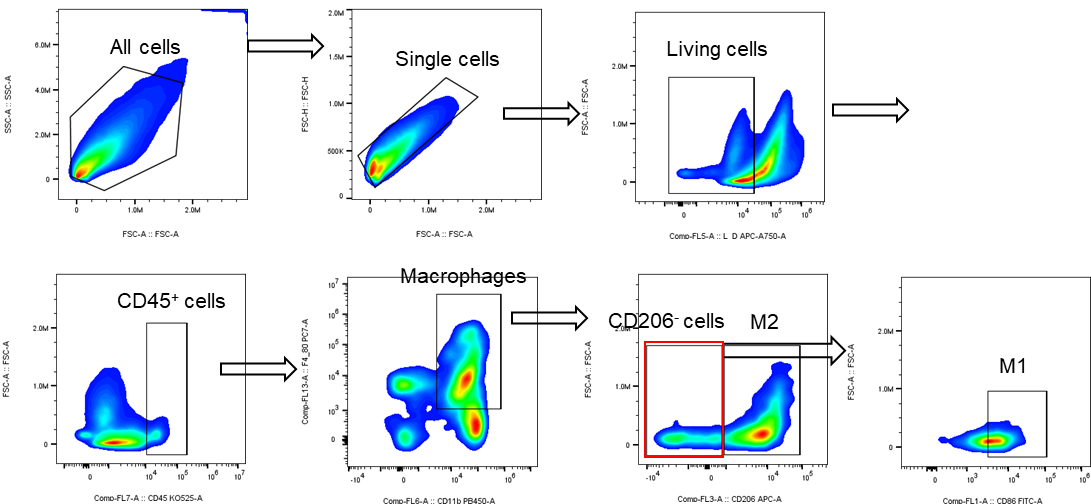


**Figure S27.** Gating strategy of M1 and M2 macrophages.


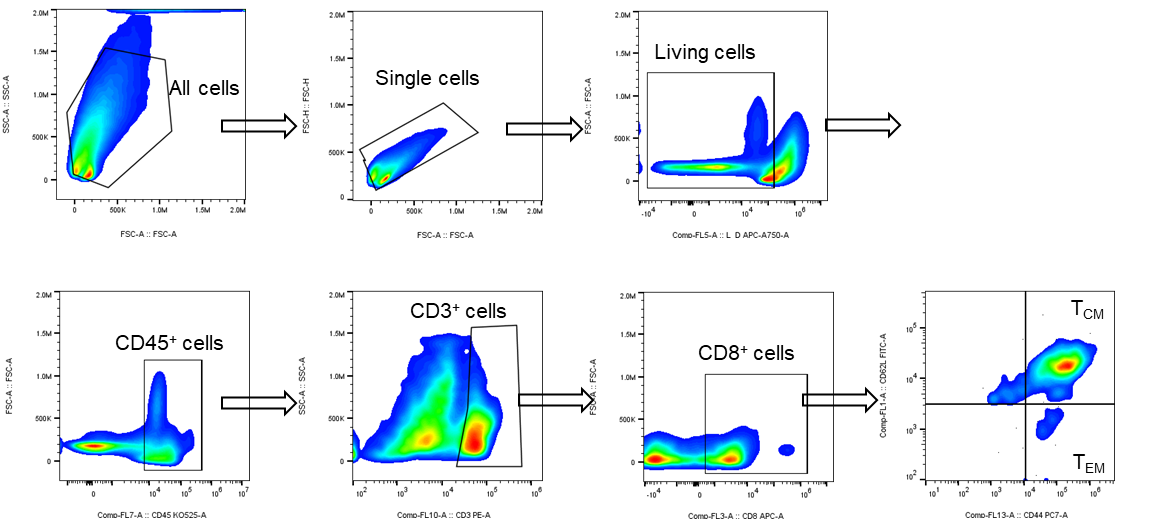


**Figure S28.** Gating strategy of T_CM_ and T_EM_ cells.


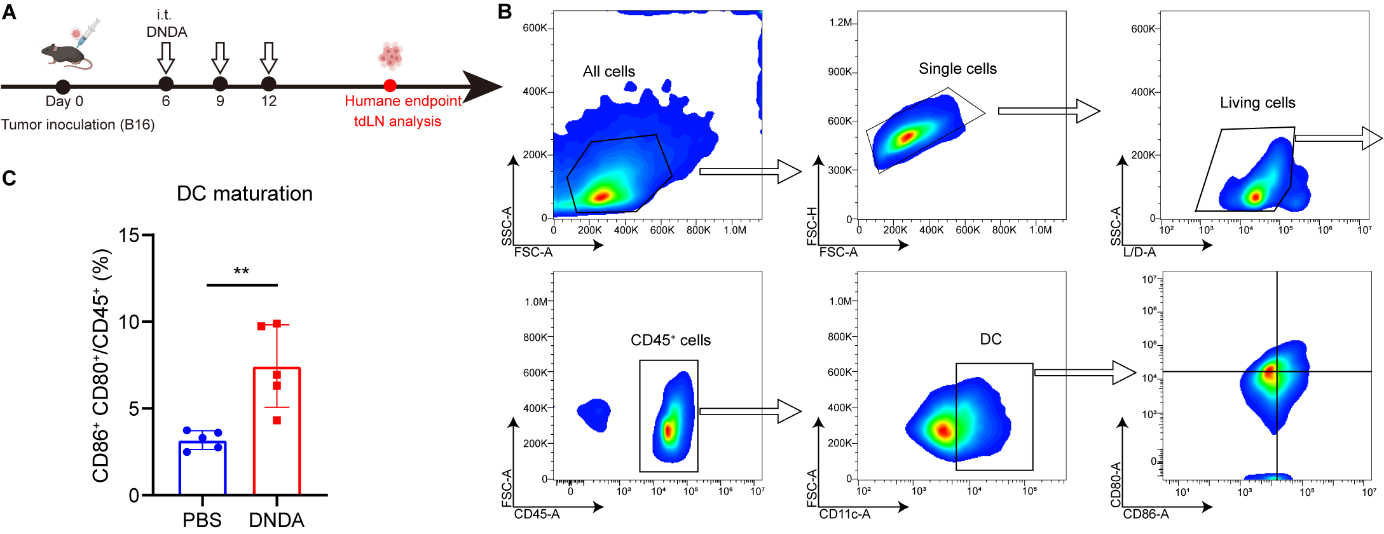


**Figure S29.** The DC maturation in tumor-draining lymph node (tdLN). (A) Treatment schedule of DNDA on B16 bearing C57BL/6 mice. (B) Gating strategy of maturated DC in tdLN. (C) Quantitative analysis of maturated DC (gated on L/D^-^ CD45^+^ CD11c^+^ CD80^+^ CD86^+^ cells) in tdLN. Data are shown as Mean ± SD (n = 5), statistical significance was calculated via two-tailed unpaired *t*-test, ***p* < 0.01.


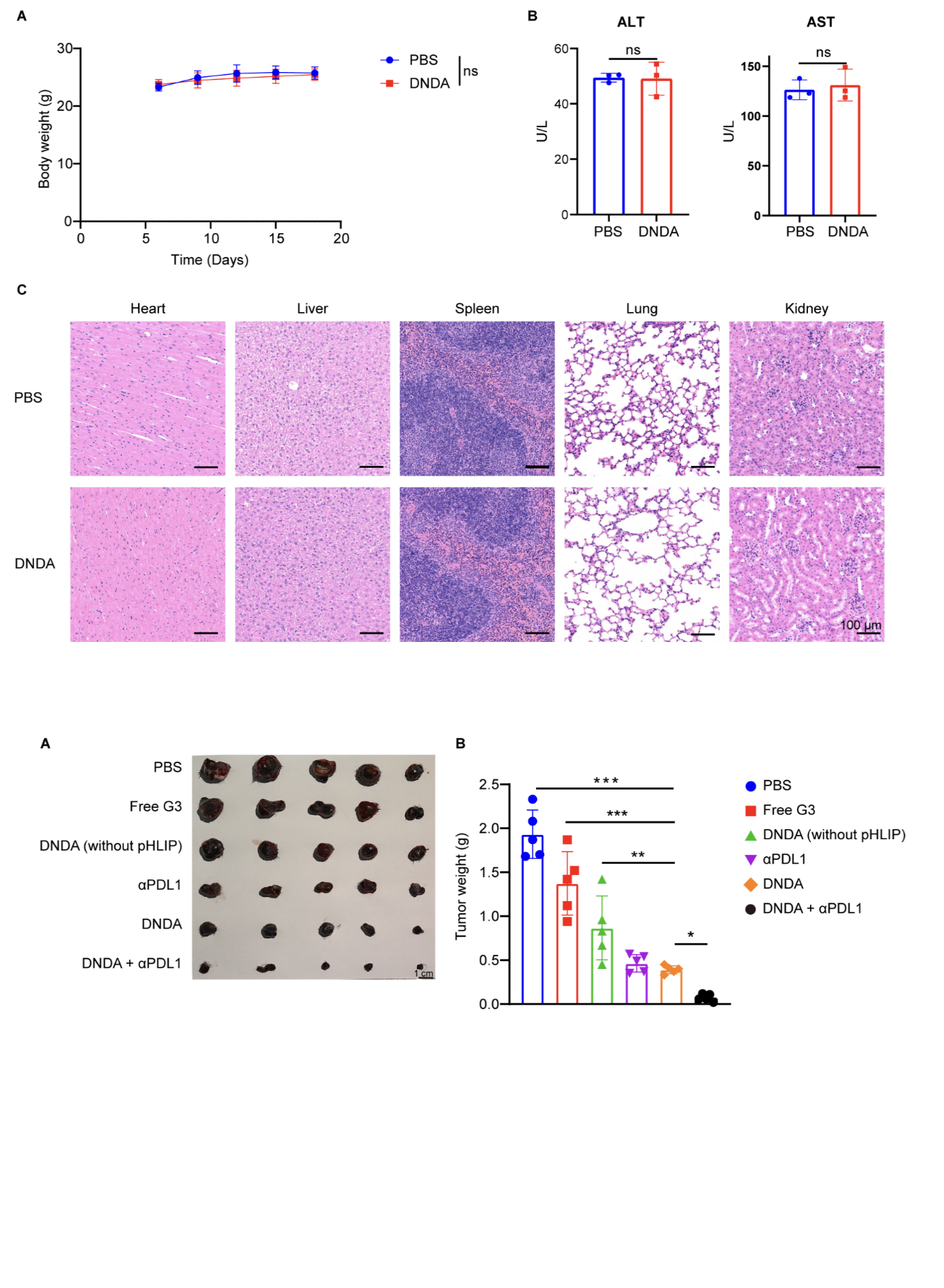


**Figure S30.** Biocompatibility analysis of DNDA in B16 tumor-bearing C57BL/6 mice. (A) Body weight measurement during the treatment cycle after intratumoral injection of DNDA or PBS. Data are shown as Mean ± SD (n = 5), statistical significance was calculated via two-way ANOVA with Tukey’s post hoc test, ns means no significance. (B) Serum biochemistry and (C) histologic analysis of major organs at 24 h post intratumoral injection of DNDA or PBS. Data are shown as Mean ± SD (n = 3), statistical significance was calculated via two-tailed unpaired *t*-test, ns means no significance.

**Table S1.** The sequence of oligonucleotides used in this study

| Name | DNA sequences (from left to right: 5’-3’) |
| --- | --- |
| Ya-1 | GACCGATGGATGACCTGTCTGCCTAATGTGCGTCGTAAG |
| Ya-1-Cy5 | GACCGATGGATGACCTGTCTGCCTAATGTGCGTCGTAAG-Cy5 |
| Ya-2 | GACCGATGGATGACTTACGACGCACAAGGAGATCATGAG |
| Ya-3 | GACCGATGGATGACTCATGATCTCCTTTAGGCAGACAGG |
| Yb-1 | TCATCCATCGGTCCCTGTCTGACTAATGTGCGTCGTAAG |
| Yb-2 | TCATCCATCGGTCCTTACGACGCACAAGGAGATCATGAG |
| Yb-3 | TCATCCATCGGTCCTCATGATCTCCTTTAGTCAGACAGG |
| Yl | GACCGATGGATGAAAAAA |
| Yl-mal | GACCGATGGATGAAAAAA-Maleimide |
| ATP-apt | TCATCCATCGGTCAAAGGCATCACCTGGGGGAGTATTGCGGAGGA AGGT |
| ATP-G3F | CCCAGGTGATGCCGGGCAATGGTCCTGCTGGAGTTCGGG |
| G3R | GGGGAACTCCAGCAGGACCATTGGGG |
| G3R-Fam | GGGGAACTCCAGCAGGACCATTGGGG-Fam |
| Yc | TCATCCATCGGTCCTTACGACGCACATTAGGCAGACAGG |

**Table S2. Blood hematologic analysis of mice**

| Item | Reference range | PBS | | DNDA |
| --- | --- | --- | --- | --- |
| WBC (10^9^/l) | 3.48-14.03 | | 7.4 ± 0.6 | 6.2 ± 0.4 |
| LYMPH (10^9^/l) | 2.22-9.83 | | 5.7 ± 0.6 | 4.6 ± 0.3 |
| MONO (10^9^/l) | 0.21-1.25 | | 0.2 ± 0.0 | 0.2 ± 0.0 |
| NEUT (10^9^/l) | 0.58-3.83 | | 1.6 ± 0.1 | 1.4 ± 0.1 |
| LYMPH (%) | 48.81-83.19 | | 75.9 ± 2.4 | 74.0 ± 0.2 |
| MONO (%) | 3.29-12.48 | | 2.9 ± 0.4 | 3.5 ± 0.4 |
| NEUT (%) | 9.86-39.11 | | 21.2 ± 2.1 | 22.5 ± 0.3 |
| RBC (10^12^/l) | 6.93-12.24 | | 9.97 ± 0.18 | 9.66 ± 0.26 |
| HGB (g/dl) | 126-205 | | 144.7 ± 1.2 | 141.3 ± 6.4 |
| HCT (%) | 42.1-68.3 | | 46.1 ± 0.8 | 44.7 ± 1.4 |
| MCV (fl) | 50.7-64.4 | | 46.2 ± 0.6 | 46.4 ± 0.2 |
| MCH (pg) | 13.2-17.6 | | 14.5 ± 0.2 | 14.6 ± 0.7 |
| MCHC (g/dl) | 233-327 | | 313.7 ± 3.2 | 316.0 ± 15.0 |
| RDW (%) | 16.9-23.4 | | 15.4 ± 0.5 | 16.9 ± 1.8 |
| PLT (10^9^/l) | 420-1698 | | 1247.7 ± 80.5 | 1198.3 ± 28.7 |
| MPV (fl) | 4.6-5.9 | | 5.5 ± 0.2 | 5.6 ± 0.2 |

Blood samples were collected from B16 tumor-bearing mice (male, 6-8 weeks) at 24 h post intratumoral injection of DNDA or PBS. Complete blood counts: Blood levels of White blood cells (WBC), Lymphocytes (LYMPH), Monocytes (MONO), Neutrophils (NEUT), Red blood cells (RBC), Hemoglobin (HGB), Hematocrit (HCT), Mean corpuscular volume (MCV), Mean corpuscular hemoglobin (MCH), Mean corpuscular hemoglobin concentration (MCHC), Red cell volume distribution width (RDW), Platelets (PLT), Mean platelet volume (MPV). Data are presented as Mean ± SD (n =3).
